# Discovery of a SHP2 Degrader with In Vivo Anti-Tumor Activity

**DOI:** 10.3390/molecules28196947

**Published:** 2023-10-06

**Authors:** Jinmin Miao, Yunpeng Bai, Yiming Miao, Zihan Qu, Jiajun Dong, Ruo-Yu Zhang, Devesh Aggarwal, Brenson A. Jassim, Quyen Nguyen, Zhong-Yin Zhang

**Affiliations:** 1 Department of Medicinal Chemistry and Molecular Pharmacology, Purdue University, West Lafayette, IN 47907, USA; miao6@purdue.edu (J.M.); bai62@purdue.edu (Y.B.); miao45@purdue.edu (Y.M.); dong226@purdue.edu (J.D.); zhang.violet16@gmail.com (R.-Y.Z.); devesh.aggarwal@astrazeneca.com (D.A.); bjassim@purdue.edu (B.A.J.); 2 Department of Chemistry, Purdue University, West Lafayette, IN 47907, USA; qu51@purdue.edu (Z.Q.); nguye492@purdue.edu (Q.N.); 3 Institute for Cancer Research, Purdue University, West Lafayette, IN 47907, USA; 4 Institute for Drug Discovery, Purdue University, West Lafayette, IN 47907, USA

**Keywords:** protein tyrosine phosphatase, SHP2, target protein degradation, PROTAC, anti-cancer agent

## Abstract

Src homology 2 domain-containing phosphatase 2 (SHP2) is an attractive target for cancer therapy due to its multifaceted roles in both tumor and immune cells. Herein, we designed and synthesized a novel series of proteolysis targeting chimeras (PROTACs) using a SHP2 allosteric inhibitor as warhead, with the goal of achieving SHP2 degradation both inside the cell and in vivo. Among these molecules, compound **P9** induces efficient degradation of SHP2 (DC_50_ = 35.2 ± 1.5 nM) in a concentration- and time-dependent manner. Mechanistic investigation illustrates that the **P9**-mediated SHP2 degradation requires the recruitment of the E3 ligase and is ubiquitination- and proteasome-dependent. **P9** shows improved anti-tumor activity in a number of cancer cell lines over its parent allosteric inhibitor. Importantly, administration of **P9** leads to a nearly complete tumor regression in a xenograft mouse model, as a result of robust SHP2 depletion and suppression of phospho-ERK1/2 in the tumor. Hence, **P9** represents the first SHP2 PROTAC molecule with excellent in vivo efficacy. It is anticipated that **P9** could serve not only as a new chemical tool to interrogate SHP2 biology but also as a starting point for the development of novel therapeutics targeting SHP2.

## 1. Introduction

Protein tyrosine phosphatases (PTP) cooperate with protein tyrosine kinases (PTK) in regulating the level of protein tyrosine phosphorylation, which is crucial in cell signal transduction. Src homology 2 domain-containing phosphatase 2 (SHP2), encoded by *PTPN11*, is a member of the PTP family [1] that regulates cellular proliferation, survival, migration, and differentiation by participating in numerous cell-signaling cascades, such as RAS-ERK1/2, PI3K-AKT and JAK-STAT pathways [2,3,4]. Germline mutations in *PTPN11* cause Noonan [5] and LEOPARD [6] syndromes, which have overlapping clinical features. Autosomal dominant activating mutations in *PTPN11* fuel excess RAS/ERK1/2 signaling that drives certain human RASopathies and cancers [7]. Somatic mutations of *PTPN11* have been found in patients with myelodysplastic syndrome (10%), juvenile acute myeloid leukemia (AML) (5%), and B-cell acute lymphoblastic leukemia (7%) [8]. *PTPN11* mutations also occur in sporadic solid tumors including lung cancer, colon cancer, neuroblastoma, and melanoma [9]. Moreover, accumulating evidence suggests that SHP2 may play an important role in immune evasion and in the T-cell programmed cell death/checkpoint pathway (PD1/PD-L1) [10,11]. Regarded as an appealing target for human cancer therapies, significant endeavors have been dedicated to the development of SHP2 inhibitors [12,13,14]. In 2016, Novartis reported the first potent, selective, and orally bioavailable SHP2 inhibitor SHP099, which targets an allosteric binding site in SHP2 and stabilizes the inactive conformation of the enzyme [15,16]. Subsequently, several allosteric SHP2 inhibitors with superior pharmaceutical properties were developed and progressed to clinical trials for treating advanced or metastatic solid tumors [17].

Despite the clinical promise of the allosteric SHP2 inhibitors, recent studies have drawn attention to the possibility of non-mutational drug resistance mechanisms [18]. Through the acute depletion of the target protein, proteolysis targeting chimeras (PROTACs) may overcome such resistance and provide an alternative and potentially more compelling approach to inhibiting SHP2. Over the past few years, the PROTAC approach has emerged as a powerful post-translational chemical technique targeting selective protein degradation [19]. A typical PROTAC molecule consists of a ligand to the protein of interest, a ligand to an E3 ligase, and a linker between the two. PROTACs induce the proximity of target proteins with E3 ubiquitin ligases to form interactions between the two, leading to ubiquitination and subsequent degradation through the proteasome system. Compared with classical small molecule inhibitors, PROTAC degraders usually feature lower doses and higher selectivity and can often overcome mutation-caused drug resistance. With regard to SHP2, Ruess et al. have revealed that knockout of the *PTPN11* gene in KRAS mutant human ductal adenocarcinoma (PDAC) cells results in a reduction in cell proliferation and *PTPN11*-knockout cells are uniquely susceptible to mitogen-activated protein kinase (MEK) inhibitors [20]. Thus, small molecule-induced degradation of SHP2 could be an effective therapeutic strategy for human cancers, particularly those carrying a KRAS-mutation. Moreover, phosphatase-independent functions of SHP2 can also be explored and targeted using the degraders as unique tools [21].

**Figure 1:**
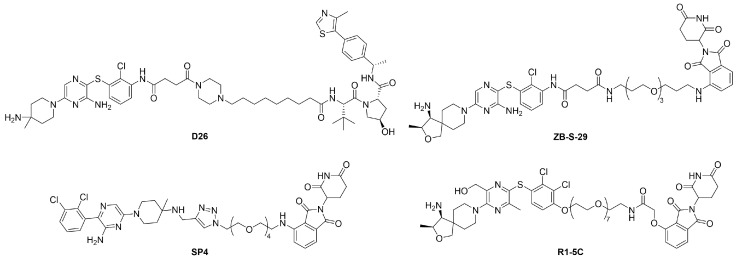
Structures of previously reported SHP2 PROTACs.

Several PROTACs that leverage the SHP2 allosteric inhibitors as ligands were identified for SHP2 degradation in cells (Figure 1) [22]. In 2020, Wang and coworkers reported the first SHP2 PROTAC D26, which recruits Von Hippel–Lindau (VHL) as the E3 ligase [23]. Shortly thereafter, several Cereblon (CRBN)-based SHP2 PROTACs including ZB-S-29 [24], SP4 [25], and R1-5C [26] were also reported. Despite having promising activities in cell culture, most of the current SHP2 PROTACs did not exhibit in vivo efficacies, although D26 did show modest anticancer activities (<20% tumor growth inhibition) as a single agent in a xenograft mice model [27]. This lack of in vivo activities limits the application of the SHP2 degraders. Herein, we describe our efforts in the discovery of a novel potent SHP2 degrader that features high efficacy in blocking tumor growth at the cellular level and in vivo in a xenograft mouse model.

## 2. Results and Discussion

### 2.1. Design of SHP2 PROTACs

**Figure 2:**
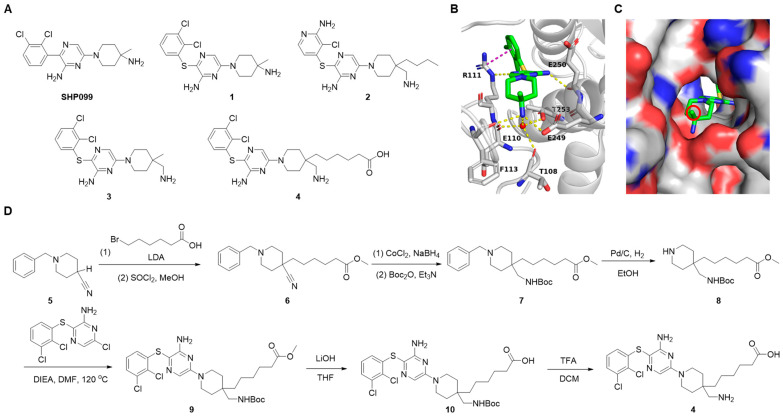
Identification of a SHP2 ligand for PROTAC development. (**A**) Chemical structures of selected SHP2 allosteric inhibitors and the designed ligand **4**. (**B**) Cocrystal structure of **1** (green stick) bound to the SHP2 allosteric pocket with the interacting structural water represented by a red sphere (PDB 7JVN). Hydrogen bonds are depicted by yellow dashes, and the cation-π interaction is shown with a purple dash. (**C**) Piperidine region of the scaffold bound to SHP2 in surface representation colored by element. The solvent-exposed methyl group exploited in PROTAC design is circled in red. (**D**) Synthetic route to the SHP2 ligand compound **4**.

The initial considerations for the rational design of PROTAC molecules include the target protein recruiting elements and the tethering site for anchoring the linkers. The co-crystal structure of SHP2 complexed with compound **1** (Figure 2A, PDB code 7JVN), a close analog of SHP099, showed that the terminal methyl group on the piperidine ring is solvent exposed and therefore is suitable for linker attachment (Figure 2B,C) [28]. Furthermore, another analogous allosteric inhibitor compound **2**, which has a longer alkyl group chain in the place of methyl group in **1**, was reported to be a potent SHP2 inhibitor (IC_50_ = 17 nM) [29]. Based on these observations, we reasoned that we could tether the linker on the terminal ethyl group on the piperidine ring in **1** without diminishing the binding affinity to SHP2. In consideration of the synthetic complexity, we chose a potent and highly cell-active SHP2 inhibitor compound **3** (SHP2 IC_50_ = 17 nM, pERK1/2 IC_50_ = 88 nM) [28] as the starting point for a ligand for SHP2 protein recruitment in this study (Figure 2A). To test this hypothesis, we designed and synthesized compound **4** which contains hexanoic acid serving as an anchor in the place of methyl group in **1** (Figure 2D). The synthesis started with the alkylation of the 1-benzylpiperidine-4-carbonitrile (**5**) with the 6-bromohexanoic acid, followed by the protection of the carboxylic acid to give the ester **6**. The reduction in this intermediate **6** with cobalt chloride and sodium borohydride and the subsequent Boc protection afforded the intermediate **7**. Removal of the piperidyl benzyl group followed by the nucleophilic substitution to a chloro-pyrazine gave the intermediate **9**, which was then converted to compound **4** by the deprotection of the carboxylic acid (intermediate **10**) and the amine. As expected, compound **4** displayed a low IC_50_ (90 nM) against full-length SHP2 in the in vitro enzymatic assay, suggesting that it is a suitable ligand for constructing SHP2 PROTAC molecules.

### 2.2. Exploration of E3 Ligands and Linkers

With the desired SHP2 ligand in hand, we synthesized an initial set of PROTAC molecules using pomalidomide or lenalidomide as a ligand of the E3 ligase Cereblon (CRBN), which is one of the most productive E3 in the development of PROTACs [30]. However, the synthesized PROTAC compounds did not induce significant degradation of SHP2 in cells. Further investigation of these PROTAC candidates revealed that they are steadily hydrolyzed and oxidized in cell mediums or in the presence of water at room temperature (Scheme S1). LCMS analysis showed that over 90% of the compounds (**11** and **12**) were converted into ring-opened products **13** within 3 h in the Dulbecco’s Modified Eagle Medium (DMEM) cell media or in water at room temperature (Scheme S1). This observation is consistent with the fact that phthalimides can be smoothly hydrolyzed in the presence of water and an organic base [31], as these PROTAC compounds contain a basic aliphatic amino group. Given that this free amino group in the allosteric inhibitors is essential for the binding affinity to SHP2 [28], we reasoned that pomalidomide or lenalidomide-based CRBN ligands are not suitable for the development of in vivo efficacious SHP2 degraders in this study due to stability considerations.

We then turned to VHL, another E3 ligase that is commonly employed in PROTAC development [32]. Since the linker length and compositions both play important roles in PROTAC design and have an essential influence on the degradation potency [33], we synthesized and evaluated an initial set of PROTACs with varied linear alkyl chains and polyethylene glycols using VHL-2 (colored in red) as the VHL ligand [34] to reveal the favorable linker length (Table 1). The biochemical enzymatic assay showed that these PROTAC candidates inhibited SHP2 with IC_50_ values of 100~200 nM, which is similar to that of compound **4** (the SHP2 ligand used for the PROTAC construction with an IC_50_ of 90 nM), indicating that installation of the linkers to compound 4 does not lead to loss of SHP2 binding (Table S1). Subsequently, these PROTAC candidates were surveyed by Western blotting in the HEK293 human embryonic kidney cell line for their ability to induce degradation of SHP2 protein at 1 and 10 µM (Table 1 and Figure S1). To our delight, compound **SC11**, which consists of a n-undecane linker between compound **4** and the VHL ligand (12-atom long), induced 27% and 64% degradation of SHP2 at 1 µM and 10 µM, respectively. Other compounds with different alkyl chain linkers (**SC5**, **SC7**, **SC9**) showed negligible to modest degradation at these concentrations, suggesting that SC11 has the most favored linker in this series. Nevertheless, compounds with polyethylene glycol linkers (**P3**, **P4**, **P5**) did not induce significant degradation of SHP2, although the linker in **P3** has the same atom numbers as that of **SC11**.

To achieve higher efficiency of SHP2 degradation, we further optimized the length and chemical composition of the linkers by synthesizing and evaluating the second set of PROTACs with a total linker length similar to that in **SC11** (Table 2 and Table S2). First, we synthesized **SC10** with a n-decane chain to make the linker length 1 atom shorter than that of **SC11**. After 16 h treatment, **SC10** induced 36% SHP2 degradation at 1 µM, suggesting that the 11-atom long carbon chain might be preferred to the 12-atom. With the aim of modulating lipophilicity, solubility, and rigidity and therefore improving the permeability of the PROTACs, we restrained the conformation of the linkers by introducing a positively charged piperazinyl group, yielding compounds **P7 P8**, **P9**, and **P10**. Western blotting data revealed that both **P7** and **P8** induce a higher degree of SHP2 degradation compared to their counter PROATCs with alkyl chain linkers (**SC10** and **SC11**), while **P9** achieved nearly complete depletion of SHP2 at 1 µM. Compound **P10** with a linker length of 14-atom appeared to be less effective than **P9**, indicating that a 13-atom length is optimal under the circumstances. Overall, the potency of **P9** is significantly higher than any other PROTACs we obtained, making it the lead compound in our study.

### 2.3. Dose- and Time-Dependency Study of ***P9***

**Figure 3:**
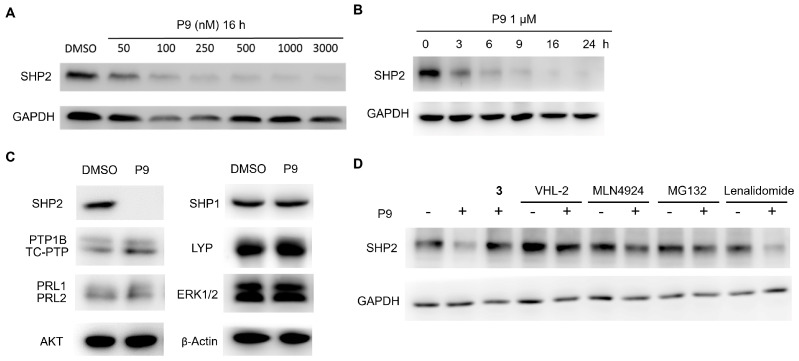
**P9** is a genuine SHP2 PROTAC degrader. (**A**) **P9** induces the degradation of SHP2 in a dose–dependent manner in HEK293 cells for 16 h of treatment. (**B**) **P9** induces degradation of SHP2 in a time-dependent manner in HEK293 cells. (**C**) **P9** treatment does not affect the protein level of other PTPs and common proteins in HEK293 cells. Cells were treated with 1 µM P9 for 16 h. (**D**) Control experiments show that **P9**–induced SHP2 degradation requires the formation of SHP2–P9–VHL ternary complex and is ubiquitination- and proteasome–dependent. HEK 293 cells were treated for 16 h with DMSO, 250 nM **P9**, or 250 nM **P9** in combination with 1 µM **3**, 10 µM MG132, 1 µM MLN4924, 40 µM VHL–2, or 40 µM Lenalidomide.

To further evaluate the ability of **P9** to induce SHP2 degradation, Western blotting was used to analyze the levels of SHP2 in HEK293 cells incubated with different concentrations of the degrader (Figure 3A). The results clearly illustrated that SHP2 protein levels were reduced in a dose-dependent manner, and quantification of the data gave the DC_50_ (concentration required for 50% degradation of protein) of 35.2 ± 1.5 nM, and no significant hook effect was observed in the tested concentration range. To determine the kinetics of **P9**-mediated degradation of SHP2 in cells, we assessed the abundance of SHP2 protein at a series of time points after the addition of the compound (Figure 3B). Over 90% of SHP2 protein was degraded within 6 h of **P9** treatment, and the maximal SHP2 depletion was achieved in 16 h.

### 2.4. Selectivity Evaluation and Mechanism of Action Study of ***P9***

To investigate the binding affinity of **P9** to SHP2 and selectivity towards other PTPs, we determined its enzymatic IC_50_s against a panel of 15 representative PTP family members, including receptor-like, nonreceptor-like, and dual-specific PTPs (Table S3). The results indicated that **P9** showed an IC_50_ of 95 nM for SHP2 and no significant inhibition towards other PTPs even at up to 10 µM **P9** concentration. Next, the selectivity of **P9** as a degrader for SHP2 over other PTP family members and different classes of enzymes was investigated (Figure 3C). Western blots showed that treatment of HEK293 cells with 1 μM **P9** for 16 h led to almost complete degradation of SHP2, whereas none of the other PTPs including SHP1, PTP1B, TC-PTP, LYP, PRL1, and PRL2, were affected. In addition, under the same conditions, **P9** did not induce any appreciable degradation of other signaling proteins including ERK1/2, AKT, and β-Actin. Taken together, the above results validated **P9** as a selective SHP2 degrader. With the aim to verify the mechanism of action for **P9**, a series of control experiments were performed in cells (Figure 3D). Western blotting analysis demonstrated the addition of an excess amount of either the SHP2 inhibitor compound **3** or VHL ligand VHL-2 prohibited **P9**-induced SHP2 degradation, indicating that simultaneous binding of the degrader to both SHP2 and VHL ligase and therefore, the formation of a ternary complex was required for the SHP2 protein degradation. Furthermore, the ubiquitination- and proteasome-dependency of the degradation was assessed by the addition of the NEDD8 E1 ubiquitin-activating enzyme inhibitor MLN-4924 and the proteasome inhibitor MG-132 to the assay conditions. Pretreatment with either MLN-4924 or MG-132 markedly reduced the extent of SHP2 degradation, demonstrating that the E1 and 26S proteasomes were indeed implicated in the degradation. In addition, the CRBN ligand lenalidomide did not affect the SHP2 degradation induced by **P9**. Collectively, these observations illustrate that **P9** is a genuine SHP2 PROTAC degrader.

### 2.5. Tumor Cell Growth Inhibition Study

**Figure 4:**
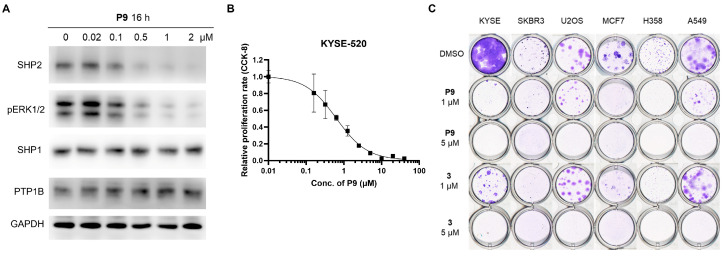
**P9** suppresses cancer cell growth by inducing degradation of SHP2. (**A**) Western blotting analysis in KYSE-520 cells after treatment with **P9** for 16 h. (**B**) CCK-8 cell proliferation assay in KYSE-520 cells with the treatment of **P9** for 7 days. (**C**) Colony formation assay of KYSE-520, SKBR3, U2OS, MCF7, H358, and A549 treated with DMSO, **P9**, or compound **3**.

SHP2 has been implicated as a major positive regulator of RTK/RAS/ERK1/2 signaling, which is aberrantly activated in a variety of human cancers [35]. SHP2 inhibitors were found to suppress the phosphorylation level of ERK1/2 and tumor growth in multiple cancer cell lines including EGFR, PI3K, and KRAS mutant cancers [17]. To determine the effect of our SHP2 degrader in a cellular context, we first evaluated the cellular effects of **P9** in squamous cell carcinoma KYSE-520 (EGFR amplified) cells [16]. Western blotting analysis showed that the SHP2 and pERK1/2 levels in KYSE-520 cells were reduced by **P9** in a dose-dependent manner, with a SHP2 degradation DC_50_ of ~130 nM and pERK1/2 inhibition EC_50_ of ~240 nM (Figure 4A). Notably, the protein levels of PTP1B and SHP1, a close homologue of SHP2, were not affected by **P9**, which further demonstrates the selectivity of the degrader over other closely related PTP family members. Consistently, cell proliferation assay indicated that **P9** restrains the growth of KYSE-520 with the IC_50_ of 0.64 ± 0.13 µM, suggesting that the PROTAC attenuates the tumor growth by degrading SHP2 and therefore inhibiting the RAS/ERK1/2 signaling pathway (Figure 4B).

We next compared the activity of **P9** to compound **3** on cancer cell growth inhibition in several cell lines previously demonstrated to be sensitive to SHP2 inhibition, including KYSE-520 (EGFR amplified) [16], SKBR3 (HER2 amplified) [36], U2OS [37], MCF7 (PIK3CA^E545K^) [36], H358 (KRAS^G12C^) and A549 (KRAS^G12S^) [36]. Compared with the parent SHP2 allosteric inhibitor compound **3**, **P9** showed improved efficacy in inhibiting the growth of these cancer cells in the colony formation assay (Figure 4C). These data suggested that **P9** is more potent than its parent SHP2 inhibitor compound **3** in blocking cell growth as measured by the colony formation assay.

### 2.6. Evaluation of ***P9*** in a Mouse Xenograft Model

**Figure 5:**
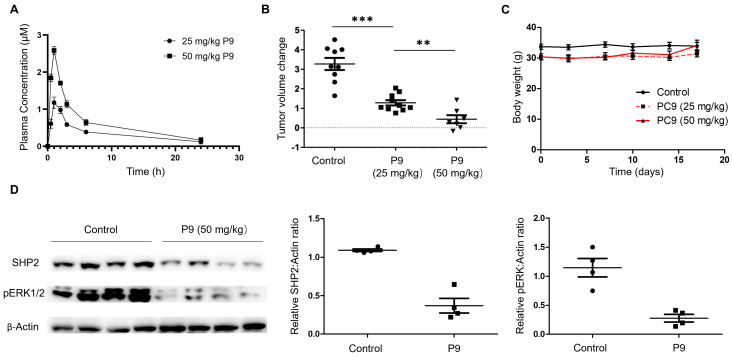
**P9** suppresses tumor growth in vivo by inducing degradation of SHP2. (**A**) Pharmacokinetic curves of **P9** in mice. (**B**) **P9** treatment dose–dependently attenuates tumor growth in a KYSE–520 xenograft model. The mice were treated with daily intraperitoneal injection of ● DMSO, ■ 25 mg/kg **P9**, or ⏷ 50 mg/kg **P9**. *** *p* ≤ 0.001. ** *p* ≤ 0.01. (**C**) **P9** treatment has no significant effect on mice body weight. (**D**) **P9** induces SHP2 degradation and decreases pERK1/2 levels in KYSE–520 tumor homogenates. ● DMSO intraperitoneal administration. ■ 50 mg/kg **P9** intraperitoneal administration.

SHP2 allosteric inhibitors have been shown to block tumor growth in mice by inhibiting the RTK/RAS/ERK1/2 signaling pathway [13,14,15,16,17,28]. To assess the antitumor activity of **P9** in vivo, we first carried out pharmacokinetics (PK) studies in mice, and the results are shown in Figure 5A. The data indicates that a single intraperitoneal injection of **P9** at 25 and 50 mg/kg achieved a peak plasma concentration (C_max_) of 1.2 ± 0.1 and 2.5 ± 0.2 µM with half-lives of 3.7 ± 0.7 and 3.0 ± 0.5 h, respectively (Tables S4 and S5). At both doses, the plasma concentrations of **P9** were maintained above 0.5 μM, which is above its effective concentration for pERK1/2 inhibition (Figure 4A), for at least 6 h. Based on the pharmacokinetic analysis, we evaluated the tolerability and antitumor efficacy of **P9** in a murine xenograft model of KYSE-520 cells exogenously. After 18 days of treatment, intraperitoneal administration of **P9** alleviated tumor progression in a dose-dependent manner as determined by serial volumetric measurement (Figure 5B). A decrease in tumor burden was observed in the SHP2 degrader **P9**-treated mice at 25 mg/kg and nearly complete tumor regression was induced following injections of **P9** at 50 mg/kg. Notably, the given compound was well tolerated in mice at the doses of 25 mg/kg or 50 mg/kg and the animal weight was preserved during the treatment procedure above (Figure 5C). Furthermore, Western blots showed that SHP2 and pERK1/2 levels are reduced to 34 ± 18% and 24 ± 12% of the control group in whole tumor homogenates after 50 mg/kg **P9** treatment, respectively (Figure 5D), indicating that the degrader attenuates the tumor growth by inducing degradation of SHP2 and inhibiting RAS/ERK1/2 signaling pathway. Taken together, the SHP2 PROTAC **P9** efficiently degrades SHP2 in the tumor tissue and effectively suppresses tumor growth in a KYSE-520 xenograft mice model. Considering that previously reported SHP2 degrader D26 alone only exerted moderate inhibition of xenograft tumor growth, **P9** represents the best SHP2 degrader with robust in vivo anti-tumor activity so far.

## 3. Materials and Methods

### 3.1. General Information

Unless otherwise noted, all reagents were purchased from commercial suppliers and used without further purification. SHP099 [15] and compound **3** [28] were prepared following the literature procedures. Thin-layer chromatography was performed using glass-precoated Merck silica gel 60 F254 plates. Flash column chromatography was performed on Biotage prepacked columns using the automated flash chromatography system Biotage Isolera One (Biotage, Uppsala, Sweden). Normal phase column chromatography was performed using KP-SIL silica gel, and reverse phase column chromatography was performed using Teledyne Isco RediSepRf Gold columns (Teledyne, Thousand Oaks, CA, USA). High-performance liquid chromatography (HPLC) purification was performed using column: Phenomenex Kinetex C18 5 μm 150 × 21.2 mm; Eluent A: water + 0.1% formic acid (99%), Eluent B: methanol; DAD scan: 210–400 nm). Melting points were measured using an *OptiMelt* MPA100 Automated Melting Point System. The ^1^H and ^13^C NMR spectra were recorded on a Bruker AVANCE 500 MHz spectrometer using chloroform-D (CDCl_3_) or dimethyl sulfoxide (DMSO-d6) as the solvents. Chemical shifts are expressed in ppm (δ scale) and referenced to the residual protonated solvent. Peak multiplicities are reported using the following abbreviations: s (singlet), d (doublet), t (triplet), q (quartet), m (multiplet), or br (broad singlet). Mass spectra and purity data were obtained using an Agilent Technologies 6470 series, triple quadrupole LC–MS (Agilent, Santa Clara, CA, USA). The purity of all final tested compounds was determined to be >95% (UV, λ = 254 nm). High-resolution mass analysis was performed on an Agilent 6550 iFunnel Q-TOF mass LC–MS. DIFMUP was purchased from Thermo Fisher Scientific (catalog# D6567, Waltham, MA, USA).

### 3.2. Synthetic Procedures

#### 3.2.1. Synthesis of SHP2 Ligand Compound **4**

A solution of 1-benzylpiperidine-4-carbonitrile (2.00 g, 10 mmol) in dry THF (17.8 mL) was prepared in a three-necked round-bottomed flask equipped with a stir bar and argon inlet adaptor. The solution was cooled to −78 °C, and a solution of lithium diisopropylamide (LDA) (1 M) in THF (10.5 mL) was added dropwise. The mixture was warmed to 0 °C, stirred for 30 min, and then cooled to −78 °C. A solution of 6-bromohexanoic acid (1.95 g, 10 mmol) in THF (20 mL) was added at −78 °C and then stirred at 0 °C for 30 min. The mixture was gradually warmed to ambient temperature and quenched with water (10 mL), and then concentrated by rotary evaporation. The residue was redissolved with MeOH, and thionyl chloride (1.6 mL, 21 mmol) was slowly added at 0 °C. The mixture was stirred at ambient temperature for 12 h, and then concentrated by rotary evaporation. The residue was purified by reverse phase flash column chromatography (60–95% gradient of methanol in water) to give methyl 6-(1-benzyl-4-cyanopiperidin-4-yl)hexanoate as a yellow solid (**6**, 1.91 g, 58%), m.p. 153.8–157.1 °C. ^1^H NMR (500 MHz, CDCl_3_) δ 7.68–7.63 (m, 2H), 7.49–7.41 (m, 3H), 3.66 (s, 3H), 3.49 (d, *J* = 12.6 Hz, 2H), 2.99–2.89 (m, 2H), 2.60–2.43 (m, 2H), 2.31 (t, *J* = 7.4 Hz, 2H), 2.04 (d, *J* = 13.4 Hz, 2H), 1.71–1.57 (m, 4H), 1.54–1.44 (m, 4H), 1.41–1.32 (m, 2H). ^13^C NMR (126 MHz, CDCl_3_) δ 173.85, 131.36, 130.47, 129.53, 127.58, 121.19, 61.25, 51.58, 49.49, 47.51, 38.50, 36.47, 33.79, 31.59, 28.75, 24.53, 24.07, 19.34. LC/MS *m*/*z* calculated [M + H]^+^ 329.22, found 329.30.

This intermediate **6** (1.64 g, 5 mol) and cobaltous chloride hexahydrate (2.74 g, 10 mmol) were dissolved in methanol (50 mL), and sodium borohydride (1.89 g, 50 mol) was added in portions while stirring at ambient temperature. The evolution of hydrogen gas was observed, and then black precipitates appeared during the addition of sodium borohydride. When the addition was complete, stirring was continued for 1 h at ambient temperature. Then di-tert-butyl dicarbonate (1.30 g, 6 mmol) and triethylamine (1.39 mL, 10 mmol) were added, and the mixture was stirred for 12 h. The mixture was then filtered through a pad of Celite with the aid of methanol (100 mL). The filtrate was concentrated by rotary evaporation and purified by reverse phase flash column chromatography (40–80% gradient of methanol in water) to give methyl 6-(1-benzyl-4-(((tert-butoxycarbonyl)amino)methyl)piperidin-4-yl)hexanoate as a pale yellow solid (**7**, 0.80 g, 37%). ^1^H NMR (500 MHz, CDCl_3_) δ 7.65 (dt, *J* = 7.7, 3.4 Hz, 2H), 7.53–7.36 (m, 3H), 3.65 (s, 3H), 3.49 (d, *J* = 12.4 Hz, 2H), 2.99–2.88 (m, 4H), 2.60–2.43 (m, 2H), 2.31 (t, *J* = 7.4 Hz, 2H), 2.04 (d, *J* = 13.4 Hz, 2H), 1.70–1.54 (m, 4H), 1.52–1.41 (m, 4H), 1.41–1.32 (m, 11H). ^13^C NMR (126 MHz, CDCl_3_) δ 173.87, 156.84, 131.71, 130.42, 129.50, 127.54, 77.95, 61.18, 51.72, 45.28, 35.67, 35.10, 34.23, 32.42, 29.97, 28.27, 24.92, 21.47. LC/MS *m*/*z* calculated [M + H]^+^ 433.31, found 433.40.

A solution of compound **7** (0.80 g, 1.85 mmol) and palladium 10% on carbon (80 mg) in ethanol (20 mL), were stirred at 70 °C for 12 h under hydrogen gas. The mixture was cooled to ambient temperature and filtered through a pad of Celite. The filtrate was concentrated by rotary evaporation, and the resulting yellow oil (compound **8**, 601 mg, 95%) was directly used in the next step without further purification. LC/MS *m*/*z* calculated [M + H]^+^ 343.26, found 343.30.

A mixture of 6-chloro-3-((2,3-dichlorophenyl)thio)pyrazin-2-amine (644 mg, 2.1 mmol) and **8** (601 mg, 1.75 mmol) in DMSO (5 mL) and DIPEA (5 mL) was stirred for 12 h at 130 °C. After cooling to RT, the volatiles were removed under reduced pressure, and the residue was purified by flash column chromatography (5–50% gradient of MeOH in DCM) to give **9** as a yellow solid (793 mg, 74%). ^1^H NMR (500 MHz, DMSO) δ 7.60 (s, 1H), 7.45–7.34 (m, 1H), 7.18 (t, *J* = 8.0 Hz, 1H), 6.80 (t, *J* = 6.5 Hz, 1H), 6.57 (dd, *J* = 8.1, 1.4 Hz, 1H), 3.66–3.56 (m, 5H), 3.55–3.46 (m, 2H), 2.94 (d, *J* = 6.3 Hz, 2H), 2.16 (t, *J* = 7.3 Hz, 2H), 1.54–1.48 (m, 2H), 1.41–1.28 (m, 13H), 1.27–1.16 (m, 6H). ^13^C NMR (126 MHz, DMSO) δ 173.94, 156.61, 156.33, 154.43, 140.39, 132.52, 128.71, 127.63, 126.95, 124.88, 120.96, 112.31, 77.92, 61.12, 51.41, 45.26, 35.39, 35.18, 33.76, 32.36, 29.80, 28.73, 25.26, 22.15. LC/MS *m*/*z* calculated [M + H]^+^ 612.20, found 612.20.

A solution of **9** (793 mg, 1.3 mmol) in a mixture of THF (10 mL) and 1 M aqueous LiOH (10 mL) was stirred at 60 °C for 4 h. After cooling to RT, the volatiles were removed under reduced pressure, and the resulting mixture was purified by reverse phase flash column chromatography (50–90% gradient of MeOH/H_2_O) to give **10** as a yellow solid (661 mg, 85%), m.p. 155.4–157.8 °C. ^1^H NMR (500 MHz, DMSO) δ ^1^H NMR (500 MHz, DMSO) δ 7.59 (s, 1H), 7.38–7.35 (m, 1H), 7.19 (t, *J* = 8.1 Hz, 1H), 6.82 (t, *J* = 6.4 Hz, 1H), 6.56 (dd, *J* = 8.1, 1.5 Hz, 1H), 3.65–3.56 (m, 2H), 3.54–3.46 (m, 2H), 2.95 (d, *J* = 6.4 Hz, 2H), 2.17 (t, *J* = 7.4 Hz, 2H), 1.53–1.47 (m, 2H), 1.41–1.29 (m, 13H), 1.27–1.16 (m, 6H). ^13^C NMR (126 MHz, DMSO) δ 175.01, 156.64, 156.37, 154.41, 140.34, 132.55, 128.76, 127.68, 126.94, 124.92, 120.92, 112.27, 77.95, 51.62, 45.25, 35.67, 35.10, 34.23, 32.42, 29.97, 28.73, 24.95, 22.36. LC/MS *m*/*z* calculated [M − H]^−^ 596.19, found 596.20.

To a solution of **10** (60 mg, 0.10 mmol) in DCM (3 mL) was added TFA (0.3 mL), and the mixture was stirred at ambient temperature for 6 h. The volatiles were removed under reduced pressure, and the residue was purified by HPLC (40–80% gradient of MeOH in 5% TFA/H_2_O) to give **4** (45 mg, 90%) as a pale-yellow solid. ^1^H NMR (500 MHz, DMSO) δ 7.60 (s, 1H), 7.44–7.34 (m, 1H), 7.18 (t, *J* = 8.1 Hz, 1H), 6.82 (t, *J* = 6.4 Hz, 1H), 6.56 (dd, *J* = 8.1, 1.5 Hz, 1H), 3.65–3.56 (m, 2H), 3.54–3.46 (m, 2H), 2.94 (d, *J* = 6.4 Hz, 2H), 2.17 (t, *J* = 7.4 Hz, 2H), 1.53–1.46 (m, 2H), 1.41–1.28 (m, 4H), 1.27–1.16 (m, 6H). ^13^C NMR (126 MHz, DMSO) δ 175.08, 156.71, 156.52, 154.49, 140.55, 132.18, 128.77, 127.76, 126.93, 124.99, 120.93, 112.28, 51.86, 48.75, 36.92, 35.71, 34.45, 32.43, 30.04, 24.98, 22.45. LC/MS *m*/*z* calculated [M + H]^+^ 498.15, found 498.20.

#### 3.2.2. Preparation of SHP2 PROTAC Linkers with E3 Ligands

**Scheme 1:**
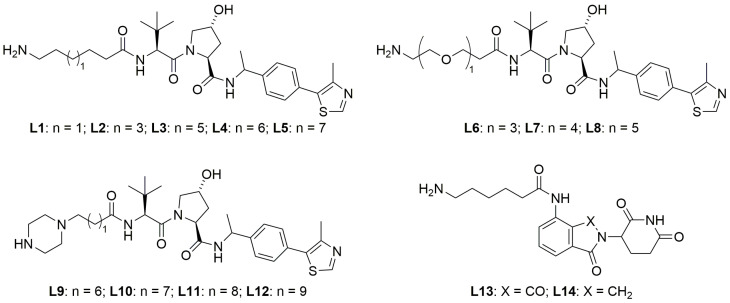
Structures of SHP2 PROTAC linkers with E3 ligands.

Compounds **L1**–**L12** (Scheme 1) were synthesized according to a published method [23]. Characterization of **L1**–**L7**, **L9**–**L11** matched well with literature. **L13** [38] and **L14** [39] (Scheme 1) were synthesized using a published method and their characterization matched well with the literature.

Characterization of **L8** as a hydrochloride salt. ^1^H NMR (500 MHz, DMSO) δ 9.03 (s, 1H), 8.48 (d, *J* = 8.0 Hz, 1H), 7.94 (brs, 3H), 7.91 (d, *J* = 8.0 Hz, 1H), 7.46−7.38 (m, 4H), 4.94−4.87 (m, 1H), 4.53 (d, *J* = 8.6 Hz, 1H), 4.47−4.40 (m, 1H), 4.27−4.20 (m, 1H), 3.67−3.45 (m, 22H), 2.97−2.92 (m, 2H), 2.46 (s, 3H), 2.39−2.32 (m, 1H), 2.08−2.03 (m, 1H), 1.86−1.79(m, 1H), 1.37 (d, *J* = 7.2 Hz, 3H), 0.94 (s, 9H). LC/MS *m*/*z* calculated [M + H]^+^ 736.40, found 736.30.

Characterization of **L11** as a hydrochloride salt. ^1^H NMR (500 MHz, DMSO) δ 8.97 (s, 1H), 8.35 (d, *J* = 7.8 Hz, 1H), 7.76 (d, *J* = 9.1 Hz, 1H), 7.45–7.35 (m, 4H), 4.94−4.87 (m, 1H), 4.50 (d, *J* = 9.1 Hz, 1H), 4.40 (t, *J* = 8.0 Hz, 1H), 4.30−4.24 (m, 1H), 3.63−3.53 (m, 3H), 3.50−3.25 (m, 6H), 3.12–3.05 (m, 2H), 2.51–2.46 (m, 3H), 2.28−2.18 (m, 1H), 2.12−2.04 (m, 1H), 2.03–1.96 (m, 1H), 1.82−1.74 (m, 1H), 1.64−1.54 (m, 2H), 1.52−1.40 (m, 2H), 1.36 (d, *J* = 7.0 Hz, 3H), 1.31–1.17 (m, 10H), 0.92 (s, 9H). ^13^C NMR (126 MHz, DMSO) δ 172.51, 171.09, 170.07, 151.98, 148.22, 145.13, 131.61, 130.17, 129.30, 126.86, 69.22, 59.02, 56.80, 56.71, 56.19, 49.06, 48.47, 48.17, 40.77, 38.22, 35.68, 35.37, 29.13, 28.92, 26.90, 26.29, 25.87, 25.42, 23.66, 22.87, 16.44. LC/MS *m*/*z* calculated [M + H]^+^ 683.43, found 683.00.

Characterization of **L12** as a hydrochloride salt. ^1^H NMR (500 MHz, DMSO) δ 8.99 (s, 1H), 8.34 (d, *J* = 8.0 Hz, 1H), 7.77 (d, *J* = 8.6 Hz, 1H), 7.48−7.37 (m, 4H), 4.96−4.90 (m, 1H), 4.51 (d, *J* = 8.6 Hz, 1H), 4.44−4.39 (m, 1H), 4.31−4.27 (m, 1H), 3.63−3.55 (m, 3H), 3.52−3.22 (m, 6H), 3.17−3.10 (m, 3H), 2.46 (s, 3H), 2.33−1.99 (m, 3H), 1.82−1.75 (m, 1H), 1.64−1.55 (m, 2H), 1.50−1.44 (m, 2H), 1.37 (d, *J* = 7.2 Hz, 3H), 1.31−1.25 (m, 12H), 0.93 (s, 9H). LC/MS *m*/*z* calculated [M + H]^+^ 697.45, found 697.50.

#### 3.2.3. Synthesis of CRBN-Based SHP2 PROTACs

**Scheme 2:**
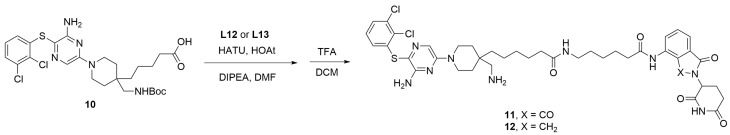
Synthesis of CRBN-based SHP2 PROTACs.

General method (Scheme 2): To a stirred solution of carboxylic acid **10** (29.9 mg, 0.05 mmol) and DIPEA (28 μL, 0.15 mmol) in DMF (2 mL) at ambient temperature was added the corresponding primary amine **L13** or **L14** (0.075 mmol) and HATU (28 mg, 0.075 mmol). The reaction mixture was stirred for 1 h. After quenched with water (5 mL) and extracted with EtOAc (5 mL × 3), the organic layers were washed with brine (15 mL), dried over anhydrous Na_2_SO_4_, and concentrated under reduced pressure. To this residue was added DCM (2 mL), and trifluoracetic acid (0.2 mL) at 0 °C. The reaction was stirred for 6 h at ambient temperature, and the volatiles were removed under reduced pressure. The residue was purified by HPLC (40–85% gradient of MeOH in 5%TFA/H_2_O) to yield the corresponding product.

6-(1-(6-amino-5-((2,3-dichlorophenyl)thio)pyrazin-2-yl)-4-(aminomethyl)piperidin-4-yl)-N-(6-((2-(2,6-dioxopiperidin-3-yl)-1,3-dioxoisoindolin-4-yl)amino)-6-oxohexyl)hexanamide (**11**) was prepared with **L13** using the general method, yield 62%, white solid. ^1^H NMR (500 MHz, DMSO) δ 9.79 (s, 1H), 8.43 (d, *J* = 8.4 Hz, 1H), 7.83 (t, *J* = 7.9 Hz, 1H), 7.64–7.57 (m, 2H), 7.44–7.34 (m, 1H), 7.18 (t, *J* = 8.1 Hz, 1H), 6.56 (dd, *J* = 8.1, 1.5 Hz, 1H), 5.15 (dd, *J* = 12.8, 5.4 Hz, 1H), 3.65–3.56 (m, 2H), 3.54–3.46 (m, 2H), 2.99–2.84 (m, 4H), 2.79–2.64 (m, 2H), 2.64–2.51 (m, 2H), 2.50–2.43 (m, 2H), 2.17–2.09 (m, 2H), 1.65–1.46 (m, 6H), 1.43–1.28 (m, 6H), 1.27–1.16 (m, 6H). LC/MS *m*/*z* calculated [M + H]^−^. 866.30, found 866.30.

6-(1-(6-amino-5-((2,3-dichlorophenyl)thio)pyrazin-2-yl)-4-(aminomethyl)piperidin-4-yl)-N-(6-((2-(2,6-dioxopiperidin-3-yl)-1-oxoisoindolin-4-yl)amino)-6-oxohexyl)hexanamide (**12**) was prepared with **L14** using the general method, yield 46%, white solid. ^1^H NMR (500 MHz, DMSO) δ ^1^H NMR (500 MHz, DMSO) δ 9.83 (s, 1H), 8.43 (d, *J* = 8.4 Hz, 1H), 7.83 (t, *J* = 7.9 Hz, 1H), 7.64–7.57 (m, 2H), 7.44–7.34 (m, 1H), 7.18 (t, *J* = 8.1 Hz, 1H), 6.56 (dd, *J* = 8.1, 1.5 Hz, 1H), 5.15 (dd, *J* = 12.8, 5.4 Hz, 1H), 4.43–4.27 (m, 2H), 3.65–3.56 (m, 2H), 3.54–3.46 (m, 2H), 2.98–2.83 (m, 4H), 2.83–2.66 (m, 2H), 2.64–2.51 (m, 2H), 2.50–2.43 (m, 2H), 2.17–2.09 (m, 2H), 1.65–1.46 (m, 6H), 1.44–1.29 (m, 6H), 1.28–1.16 (m, 6H). LC/MS *m*/*z* calculated [M + H]^+^ 852.32, found 852.30.

#### 3.2.4. Synthesis of VHL-Based SHP2 PROTACs

**Scheme 3:**
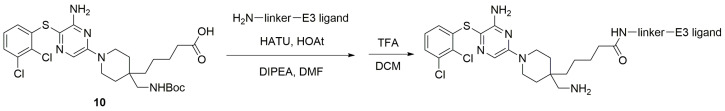
Synthesis of VHL-based SHP2 PROTACs.

General method (Scheme 3): To a stirred solution of carboxylic acid **10** (29.9 mg, 0.05 mmol) and DIPEA (28 μL, 0.15 mmol) in DMF (2 mL) at ambient temperature was added the corresponding primary amine (0.075 mmol) and HATU (28 mg, 0.075 mmol). The reaction mixture was stirred for 1 h. After quenched with water (5 mL) and extracted with EtOAc (5 mL × 3), the organic layers were washed with brine (15 mL), dried over anhydrous Na_2_SO_4_, and concentrated under reduced pressure. To this residue was added DCM (2 mL), and trifluoracetic acid (0.5 mL) at 0 °C. The reaction was stirred for 6 h at ambient temperature, and the volatiles were removed under reduced pressure. The residue was purified by HPLC (40–90% gradient of MeOH in 5%TFA/H_2_O) to yield the corresponding product.

(2S,4R)-1-((S)-2-(6-(6-(1-(6-amino-5-((2,3-dichlorophenyl)thio)pyrazin-2-yl)-4-(aminomethyl)piperidin-4-yl)hexanamido)hexanamido)-3,3-dimethylbutanoyl)-4-hydroxy-N-((S)-1-(4-(4-methylthiazol-5-yl)phenyl)ethyl)pyrrolidine-2-carboxamide (**SC5**) was prepared with **L1** using the general method, yield 48%, yellow solid. ^1^H NMR (500 MHz, DMSO) δ 9.06 (s, 1H), 8.43(d, *J* =8.0 Hz, 1H), 7.82 (d, *J* = 8.7 Hz, 1H), δ 7.60 (s, 1H), 7.53−7.34 (m, 5H), 7.17 (t, *J* = 8.1 Hz, 1H), 6.55 (dd, *J* = 8.0, 1.4 Hz, 1H), 4.95−4.89 (m, 1H), 4.51 (d, *J* = 8.8 Hz, 1H), 4.44−4.40 (m, 1H), 4.29−4.27 (m, 1H), 3.66−3.55 (m, 3H), 3.53–3.45 (m, 2H), 2.91 (d, *J* = 6.5 Hz, 2H), 2.76−2.70 (m, 2H), 2.46 (s, 3H), 2.28−2.09 (m, 4H), 2.06−1.98 (m, 1H), 1.81−1.74 (m, 1H), 1.59–1.42 (m, 7H), 1.41–1.16 (m, 15H), 0.93 (s, 9H). ^13^C NMR (126 MHz, DMSO) δ 171.56, 171.14, 171.09, 169.97, 156.34, 154.37, 152.41, 147.49, 145.35, 132.64, 131.98, 129.81, 129.30, 128.75, 127.82, 126.90, 124.95, 120.89, 117.69, 115.36, 112.71, 69.21, 59.04, 56.86, 56.72, 51.44, 51.08, 48.16, 44.28, 42.29, 38.77, 38.44, 38.18, 36.83, 35.67, 34.54, 33.74, 32.40, 31.86, 29.88, 27.02, 26.92, 22.90, 22.78, 22.64, 22.42, 16.13. LC/MS *m*/*z* calculated [M + H]^+^ 1037.44, found 1037.40.

(2S,4R)-1-((S)-2-(8-(6-(1-(6-amino-5-((2,3-dichlorophenyl)thio)pyrazin-2-yl)-4-(aminomethyl)piperidin-4-yl)hexanamido)octanamido)-3,3-dimethylbutanoyl)-4-hydroxy-N-((S)-1-(4-(4-methylthiazol-5-yl)phenyl)ethyl)pyrrolidine-2-carboxamide (**SC7**) was prepared with **L2** using the general method, yield 45%, yellow solid. ^1^H NMR (500 MHz, DMSO) δ 9.07 (s, 1H), 8.43(d, *J* =8.0 Hz, 1H), 7.82 (d, *J* = 8.7 Hz, 1H), δ 7.60 (s, 1H), 7.47−7.34 (m, 5H), 7.17 (t, *J* = 8.1 Hz, 1H), 6.55 (dd, *J* = 8.0, 1.4 Hz, 1H), 4.95−4.89 (m, 1H), 4.51 (d, *J* = 8.8 Hz, 1H), 4.44−4.40 (m, 1H), 4.29−4.25 (m, 1H), 3.62−3.55 (m, 3H), 3.53–3.45 (m, 2H), 2.91 (d, *J* = 6.5 Hz, 2H), 2.77−2.71 (m, 2H), 2.46 (s, 3H), 2.28−2.08 (m, 4H), 2.05−1.98 (m, 1H), 1.81−1.74 (m, 1H), 1.54–1.43 (m, 7H), 1.41–1.16 (m, 19H), 0.93 (s, 9H). ^13^C NMR (126 MHz, DMSO) δ 171.54, 171.13, 171.08, 169.92, 156.32, 154.34, 152.40, 147.45, 145.32, 132.62, 131.94, 129.75, 129.25, 128.74, 127.81, 126.86, 124.92, 120.87, 117.66, 115.34, 112.70, 69.20, 59.03, 56.83, 56.71, 51.41, 51.05, 48.15, 44.24, 42.27, 38.75, 38.42, 38.17, 36.68, 35.63, 35.41, 33.71, 32.38, 31.84, 29.87, 26.93, 26.88, 22.90, 22.75, 22.66, 22.47, 22.41, 22.23, 16.10. LC/MS *m*/*z* calculated [M + H]^+^ 1065.47, found 1065.40.

(2S,4R)-1-((S)-2-(10-(6-(1-(6-amino-5-((2,3-dichlorophenyl)thio)pyrazin-2-yl)-4-(aminomethyl)piperidin-4-yl)hexanamido)decanamido)-3,3-dimethylbutanoyl)-4-hydroxy-N-((S)-1-(4-(4-methylthiazol-5-yl)phenyl)ethyl)pyrrolidine-2-carboxamide (**SC9**) was prepared with **L3** using the general method, yield 50%, yellow solid. ^1^H NMR (500 MHz, DMSO) δ 9.06 (s, 1H), 8.42 (d, *J* =8.0 Hz, 1H), 7.79 (d, *J* = 8.8 Hz, 1H), δ 7.60 (s, 1H), 7.48−7.34 (m, 5H), 7.16 (t, *J* = 8.1 Hz, 1H), 6.55 (dd, *J* = 8.0, 1.4 Hz, 1H), 4.95−4.87 (m, 1H), 4.51 (d, *J* = 8.8 Hz, 1H), 4.42−4.39 (m, 1H), 4.29−4.25 (m, 1H), 3.63−3.55 (m, 3H), 3.53–3.45 (m, 2H), 3.15−3.08 (m, 1H), 2.91 (d, *J* = 6.5 Hz, 2H), 2.76−2.69 (m, 2H), 2.46 (s, 3H), 2.28−2.06 (m, 4H), 2.04−1.98 (m, 1H), 1.81−1.74 (m, 1H), 1.54–1.43 (m, 7H), 1.41–1.16 (m, 23H), 0.93 (s, 9H). ^13^C NMR (126 MHz, DMSO) δ 171.53, 171.12, 171.07, 169.91, 156.32, 154.33, 152.39, 147.45, 145.31, 132.62, 131.92, 129.74, 129.23, 128.74, 127.80, 126.83, 124.91, 120.87, 117.65, 115.33, 112.70, 69.19, 59.01, 56.76, 56.70, 51.36, 51.01, 48.15, 44.22, 42.23, 38.66, 38.40, 38.17, 36.64, 35.63, 35.25, 33.69, 32.37, 31.84, 29.85, 26.85, 26.76, 22.88, 22.69, 22.51, 22.39, 22.37, 22.18, 22.06, 21.97, 16.07. LC/MS *m*/*z* calculated [M + H]^+^ 1093.50, found 1093.50.

(2S,4R)-1-((S)-2-(12-(6-(1-(6-amino-5-((2,3-dichlorophenyl)thio)pyrazin-2-yl)-4-(aminomethyl)piperidin-4-yl)hexanamido)dodecanamido)-3,3-dimethylbutanoyl)-4-hydroxy-N-((S)-1-(4-(4-methylthiazol-5-yl)phenyl)ethyl)pyrrolidine-2-carboxamide (**SC11**) was prepared with **L5** using the general method, yield 41%, yellow solid. ^1^H NMR (500 MHz, DMSO) δ 9.00 (s, 1H), 8.37 (d, *J* =8.0 Hz, 1H), 7.79 (d, *J* = 8.8 Hz, 1H), δ 7.59 (s, 1H), 7.46−7.33 (m, 5H), 7.15 (t, *J* = 8.1 Hz, 1H), 6.55 (dd, *J* = 8.0, 1.4 Hz, 1H), 4.95−4.88 (m, 1H), 4.51 (d, *J* = 8.8 Hz, 1H), 4.42−4.39 (m, 1H), 4.29−4.26 (m, 1H), 3.65−3.56 (m, 3H), 3.53–3.45 (m, 2H), 3.16−3.09 (m, 1H), 2.90 (d, *J* = 6.4 Hz, 2H), 2.79−2.71 (m, 2H), 2.45 (s, 3H), 2.32−1.98 (m, 5H), 1.82−1.75 (m, 1H), 1.51–1.43 (m, 7H), 1.41–1.16 (m, 27H), 0.93 (s, 9H). ^13^C NMR (126 MHz, DMSO) δ 171.51, 171.09, 171.06, 169.90, 156.29, 154.30, 152.32, 147.44, 145.30, 132.60, 131.88, 129.71, 129.21, 128.72, 127.79, 126.80, 124.88, 120.86, 117.64, 115.33, 112.69, 69.16, 58.95, 56.73, 56.65, 51.33, 51.01, 48.12, 44.20, 42.18, 38.61, 38.38, 38.12, 36.42, 35.60, 35.18, 33.65, 32.32, 31.81, 29.82, 26.77, 26.68, 22.84, 22.52, 22.43, 22.38, 22.30, 22.11, 21.98, 21.89, 21.81, 21.75, 16.04. LC/MS *m*/*z* calculated [M + H]^+^ 1121.54, found 1121.50.

(2S,4R)-1-((S)-22-(1-(6-amino-5-((2,3-dichlorophenyl)thio)pyrazin-2-yl)-4-(aminomethyl)piperidin-4-yl)-2-(tert-butyl)-4,17-dioxo-7,10,13-trioxa-3,16-diazadocosanoyl)-4-hydroxy-N-((S)-1-(4-(4-methylthiazol-5-yl)phenyl)ethyl)pyrrolidine-2-carboxamide (**P3**) was prepared with **L6** using the general method, yield 55%, yellow solid. ^1^H NMR (500 MHz, DMSO) δ 9.10 (s, 1H), 8.42 (d, *J* = 8.0 Hz, 1H), 7.88 (d, *J* = 8.0 Hz, 1H), 7.61 (s, 1H), 7.46–7.34 (m, 5H), 7.18 (t, *J* = 8.1 Hz, 1H), 6.57 (dd, *J* = 8.1, 1.5 Hz, 1H), 4.95–4.88 (m, 1H), 4.52 (d, *J* = 8.8 Hz, 1H), 4.44–4.40 (m, 1H), 4.29–4.26 (m, 1H), 3.65–3.46 (m, 17H), 2.97–2.91 (m, 3H), 2.46 (s, 3H), 2.39–2.33 (m, 1H), 2.17 (t, *J* = 7.4 Hz, 2H), 2.05–2.00 (m, 1H), 1.81–1.74 (m, 1H), 1.53–1.45 (m, 2H), 1.42–1.28 (m, 7H), 1.27–1.16 (m, 6H), 0.94 (s, 9H). ^13^C NMR (126 MHz, DMSO) δ 173.01, 171.42, 171.09, 169.95, 156.35, 154.36, 152.37, 147.49, 145.36, 132.61, 131.96, 129.81, 129.30, 128.74, 127.78, 126.88, 124.92, 120.91, 117.67, 115.35, 112.70, 71.96, 71.53, 71.34, 71.21, 71.08, 70.92, 69.21, 59.01, 56.83, 56.78, 51.48, 51.07, 48.66, 44.43, 42.28, 38.84, 38.49, 38.21, 35.70, 34.60, 34.03, 33.72, 32.38, 31.85, 29.90, 26.94, 22.92, 22.43, 16.54. LC/MS *m*/*z* calculated [M + H]^+^ 1127.47, found 1127.50.

(2S,4R)-1-((S)-25-(1-(6-amino-5-((2,3-dichlorophenyl)thio)pyrazin-2-yl)-4-(aminomethyl)piperidin-4-yl)-2-(tert-butyl)-4,20-dioxo-7,10,13,16-tetraoxa-3,19-diazapentacosanoyl)-4-hydroxy-N-((S)-1-(4-(4-methylthiazol-5-yl)phenyl)ethyl)pyrrolidine-2-carboxamide (**P4**) was prepared with **L7** using the general method, yield 51%, yellow solid. ^1^H NMR (500 MHz, DMSO) δ 9.05 (s, 1H), 8.46 (d, *J* = 8.0 Hz, 1H), 7.61 (s, 1H), 7.45–7.34 (m, 5H), 7.18 (t, *J* = 8.1 Hz, 1H), 6.56 (dd, *J* = 8.1, 1.5 Hz, 1H), 4.94–4.87 (m, 1H), 4.53 (d, *J* = 8.8 Hz, 1H), 4.46–4.41 (m, 1H), 4.31–4.27 (m, 1H), 3.65–3.46 (m, 20H), 2.98–2.91 (m, 2H), 2.46 (s, 3H), 2.39–2.33 (m, 1H), 2.17 (t, *J* = 7.4 Hz, 2H), 2.08–2.03 (m, 1H), 1.82–1.74 (m, 1H), 1.53–1.45 (m, 2H), 1.43–1.27 (m, 7H), 1.27–1.15 (m, 6H), 0.94 (s, 9H). ^13^C NMR (126 MHz, DMSO) δ 173.02, 171.45, 171.12, 169.97, 156.36, 154.37, 152.39, 147.49, 145.37, 132.63, 131.97, 129.82, 129.31, 128.74, 127.79, 126.90, 124.93, 120.91, 117.67, 115.36, 112.72, 71.39, 71.35, 71.28, 71.23, 71.21, 71.14, 70.93, 69.21, 68.35, 59.06, 56.92, 56.86, 51.49, 51.09, 48.72, 44.45, 42.31, 38.95, 38.51, 38.24, 35.73, 34.65, 34.06, 33.75, 32.39, 31.86, 29.90, 26.96, 22.95, 22.47, 16.58. LC/MS *m*/*z* calculated [M + H]^+^.1171.50, found 1171.50.

(2S,4R)-1-((S)-28-(1-(6-amino-5-((2,3-dichlorophenyl)thio)pyrazin-2-yl)-4-(aminomethyl)piperidin-4-yl)-2-(tert-butyl)-4,23-dioxo-7,10,13,16,19-pentaoxa-3,22-diazaoctacosanoyl)-4-hydroxy-N-((S)-1-(4-(4-methylthiazol-5-yl)phenyl)ethyl)pyrrolidine-2-carboxamide (**P5**) was prepared with **L8** using the general method, yield 56%, yellow solid. ^1^H NMR (500 MHz, DMSO) δ 9.03 (s, 1H), 8.45 (d, *J* = 8.0 Hz, 1H), 7.61 (s, 1H), 7.44–7.34 (m, 5H), 7.18 (t, *J* = 8.1 Hz, 1H), 6.56 (dd, *J* = 8.1, 1.5 Hz, 1H), 4.95–4.87 (m, 1H), 4.53 (d, *J* = 8.8 Hz, 1H), 4.46–4.41 (m, 1H), 4.31–4.27 (m, 1H), 3.65–3.46 (m, 23H), 2.98–2.91 (m, 3H), 2.46 (s, 3H), 2.39–2.33 (m, 1H), 2.17 (t, *J* = 7.4 Hz, 2H), 2.09–2.03 (m, 1H), 1.83–1.75 (m, 1H), 1.54–1.45 (m, 2H), 1.43–1.27 (m, 7H), 1.27–1.16 (m, 6H), 0.94 (s, 9H). ^13^C NMR (126 MHz, DMSO) δ 173.05, 171.48, 171.13, 169.98, 156.36, 154.38, 152.40, 147.51, 145.39, 132.65, 131.98, 129.83, 129.32, 128.74, 127.82, 126.91, 124.94, 120.91, 117.68, 115.37, 112.74, 71.17, 71.08, 71.02, 70.78, 70.54, 70.33, 70.25, 69.84, 69.32, 68.16, 67.75 59.07, 56.95, 56.88, 51.49, 51.11, 48.73, 44.47, 42.44, 38.99, 38.51, 38.27, 35.76, 34.69, 34.06, 33.76, 32.39, 31.90, 29.93, 26.97, 22.96, 22.48, 16.63. LC/MS *m*/*z* calculated [M + H]^+^ 1215.53, found 1215.60.

(2S,4R)-1-((S)-2-(11-(6-(1-(6-amino-5-((2,3-dichlorophenyl)thio)pyrazin-2-yl)-4-(aminomethyl)piperidin-4-yl)hexanamido)undecanamido)-3,3-dimethylbutanoyl)-4-hydroxy-N-((S)-1-(4-(4-methylthiazol-5-yl)phenyl)ethyl)pyrrolidine-2-carboxamide (**SC10**) was prepared with **L4** using the general method, yield 40%, yellow solid. ^1^H NMR (500 MHz, DMSO) δ 9.02 (s, 1H), 8.39 (d, *J* =8.0 Hz, 1H), 7.79 (d, *J* = 8.8 Hz, 1H), δ 7.60 (s, 1H), 7.48−7.34 (m, 5H), 7.16 (t, *J* = 8.1 Hz, 1H), 6.55 (dd, *J* = 8.0, 1.4 Hz, 1H), 4.95−4.87 (m, 1H), 4.51 (d, *J* = 8.8 Hz, 1H), 4.42−4.38 (m, 1H), 4.27−4.24 (m, 1H), 3.63−3.55 (m, 3H), 3.53–3.45 (m, 2H), 3.16−3.09 (m, 1H), 2.90 (d, *J* = 6.4 Hz, 2H), 2.77−2.71 (m, 2H), 2.46 (s, 3H), 2.28−2.08 (m, 4H), 2.02−1.96 (m, 1H), 1.82−1.76 (m, 1H), 1.54–1.43 (m, 7H), 1.41–1.15 (m, 25H), 0.93 (s, 9H). ^13^C NMR (126 MHz, DMSO) δ 171.53, 171.11, 171.07, 169.90, 156.30, 154.31, 152.35, 147.45, 145.31, 132.60, 131.90, 129.72, 129.21, 128.73, 127.80, 126.82, 124.89, 120.87, 117.64, 115.33, 112.69, 69.18, 58.98, 56.74, 56.66, 51.35, 51.01, 48.14, 44.21, 42.20, 38.62, 38.39, 38.16, 36.43, 35.61, 35.21, 33.68, 32.36, 31.83, 29.84, 26.82, 26.71, 22.86, 22.63, 22.47, 22.38, 22.34, 22.15, 22.01, 21.92, 21.86, 16.05. LC/MS *m*/*z* calculated [M + H]^+^, 1107.52, found 1107.50.

(2S,4R)-1-((S)-2-(8-(4-(6-(1-(6-amino-5-((2,3-dichlorophenyl)thio)pyrazin-2-yl)-4-(aminomethyl)piperidin-4-yl)hexanoyl)piperazin-1-yl)octanamido)-3,3-dimethylbutanoyl)-4-hydroxy-N-((S)-1-(4-(4-methylthiazol-5-yl)phenyl)ethyl)pyrrolidine-2-carboxamide (**P7**) was prepared with **L9** using the general method, yield 47%, yellow solid. ^1^H NMR (500 MHz, DMSO) δ 8.96 (s, 1H), 8.34 (d, *J* = 7.8 Hz, 1H), 7.82–7.73 (m, 3H), 7.62 (s, 1H), 7.47–7.34 (m, 4H), 7.20 (t, *J* = 7.9 Hz, 1H), 6.55 (dd, *J* = 8.1, 1.5 Hz, 1H), 4.93−4.86 (m, 1H), 4.51 (d, *J* = 9.4 Hz, 1H), 4.43–4.36 (m, 1H), 4.29–4.24 (m, 1H), 3.67–3.29 (m, 9H), 3.09– 3.01 (m, 2H), 2.99–2.81 (m, 5H), 2.44 (s, 3H), 2.36–2.29 (m, 2H), 2.28–2.20 (m, 1H), 2.14–2.06 (m, 1H), 2.04–1.96 (m, 1H), 1.81−1.75 (m, 1H), 1.65–1.55 (m, 2H), 1.55–1.32 (m, 12H), 1.30–1.17 (m, 10H), 0.92 (s, 9H). ^13^C NMR (126 MHz, DMSO) δ 172.58, 171.48, 171.14, 170.17, 156.43, 154.41, 152.04, 148.27, 145.24, 132.66, 131.73, 130.17, 129.43, 128.82, 127.87, 126.93, 124.98, 121.94, 117.73, 115.37, 112.77, 69.34, 59.21, 56.89, 57.78, 56.17, 51.49, 51.12, 48.32, 44.31, 42.42, 38.56, 38.41, 36.89, 35.53, 34.28, 33.86, 32.44, 31.97, 29.96, 29.48, 29.22, 27.31, 27.78, 26.39, 22.95, 22.46, 16.53. LC/MS *m*/*z* calculated [M + H]^+^, 1134.53, found 1134.50.

(2S,4R)-1-((S)-2-(9-(4-(6-(1-(6-amino-5-((2,3-dichlorophenyl)thio)pyrazin-2-yl)-4-(aminomethyl)piperidin-4-yl)hexanoyl)piperazin-1-yl)nonanamido)-3,3-dimethylbutanoyl)-4-hydroxy-N-((S)-1-(4-(4-methylthiazol-5-yl)phenyl)ethyl)pyrrolidine-2-carboxamide (**P8**) was prepared with **L10** using the general method, yield 49%, yellow solid. ^1^H NMR (500 MHz, DMSO) δ 8.96 (s, 1H), 8.35 (d, *J* = 7.8 Hz, 1H), 7.82–7.73 (m, 3H), 7.62 (s, 1H), 7.46–7.34 (m, 4H), 7.19 (t, *J* = 8.0 Hz, 1H), 6.55 (dd, *J* = 8.1, 1.5 Hz, 1H), 4.93−4.86 (m, 1H), 4.51 (d, *J* = 9.4 Hz, 1H), 4.43–4.36 (m, 1H), 4.29–4.24 (m, 1H), 3.67–3.29 (m, 9H), 3.09–3.01 (m, 2H), 2.99– 2.81 (m, 5H), 2.44 (s, 3H), 2.36–2.30 (m, 2H), 2.29–2.20 (m, 1H), 2.14–2.06 (m, 1H), 2.04–1.96 (m, 1H), 1.81−1.75 (m, 1H), 1.65–1.55 (m, 2H), 1.55–1.32 (m, 12H), 1.30–1.17 (m, 12H), 0.92 (s, 9H). ^13^C NMR (126 MHz, DMSO) δ 172.54, 171.46, 171.12, 170.11, 156.35, 154.36, 152.02, 148.22, 145.18, 132.62, 131.67, 130.15, 129.32, 128.76, 127.83, 126.88, 124.94, 120.91, 117.69, 115.36, 112.72, 69.27, 59.10, 56.84, 56.77, 56.11, 51.44, 51.08, 48.22, 44.26, 42.29, 38.47, 38.26, 35.83, 35.45, 34.22, 33.79, 32.40, 31.91, 29.89, 29.35, 29.14, 27.16, 26.61, 26.23, 25.17, 22.89, 22.43, 16.48. LC/MS *m*/*z* calculated [M + H]^+^, 1148.55, found 1148.50.

(2S,4R)-1-((S)-2-(10-(4-(6-(1-(6-amino-5-((2,3-dichlorophenyl)thio)pyrazin-2-yl)-4-(aminomethyl)piperidin-4-yl)hexanoyl)piperazin-1-yl)decanamido)-3,3-dimethylbutanoyl)-4-hydroxy-N-((S)-1-(4-(4-methylthiazol-5-yl)phenyl)ethyl)pyrrolidine-2-carboxamide (**P9**) was prepared with **L11** using the general method, yield 50%, yellow solid, m.p. 207.2–211.6 °C. ^1^H NMR (500 MHz, DMSO) δ 8.97 (s, 1H), 8.35 (d, *J* = 7.8 Hz, 1H), 7.93 (s, 2H), 7.82–7.73 (m, 3H), 7.62 (s, 1H), 7.45–7.34 (m, 4H), 7.19 (t, *J* = 8.0 Hz, 1H), 6.55 (dd, *J* = 8.1, 1.4 Hz, 1H), 4.93−4.86 (m, 1H), 4.50 (d, *J* = 9.4 Hz, 1H), 4.45–4.36 (m, 1H), 4.28–4.24 (m, 1H), 4.09–3.99 (m, 1H), 3.67–3.30 (m, 9H), 3.09–3.02 (m, 2H), 2.99–2.81 (m, 5H), 2.44 (s, 3H), 2.36–2.30 (m, 2H), 2.29–2.19 (m, 1H), 2.13–2.05 (m, 1H), 2.03–1.96 (m, 1H), 1.81−1.74 (m, 1H), 1.65–1.56 (m, 2H), 1.54–1.33 (m, 12H), 1.30–1.16 (m, 14H), 0.92 (s, 9H). ^13^C NMR (126 MHz, DMSO) δ 172.49, 171.42, 171.09, 170.06, 156.33, 154.35, 151.98, 148.19, 145.12, 132.62, 131.61, 130.16, 129.30, 128.73, 127.79, 126.86, 124.92, 120.89, 117.68, 115.35, 112.70, 69.22, 59.02, 56.78, 56.71, 56.05, 51.44, 51.06, 48.16, 44.26, 42.27, 38.42, 38.23, 35.68, 35.37, 34.04, 33.73, 32.38, 31.84, 29.84, 29.13, 28.91, 26.90, 26.38, 25.88, 24.98, 23.57, 22.87, 22.41, 16.43. LC/MS *m*/*z* calculated [M + H]^+^ 1162.56, found 1162.70. HRMS *m*/*z* [M + H]^+^ Calcd for C_59_H_86_Cl_2_N_11_O_5_S_2_ 1162.5627; found 1162.5635.

(2S,4R)-1-((S)-2-(11-(4-(6-(1-(6-amino-5-((2,3-dichlorophenyl)thio)pyrazin-2-yl)-4-(aminomethyl)piperidin-4-yl)hexanoyl)piperazin-1-yl)undecanamido)-3,3-dimethylbutanoyl)-4-hydroxy-N-((S)-1-(4-(4-methylthiazol-5-yl)phenyl)ethyl)pyrrolidine-2-carboxamide (**P10**) was prepared with **L12** using the general method, yield 44%, yellow solid. ^1^H NMR (500 MHz, DMSO) δ 8.98 (s, 1H), 8.35 (d, *J* = 7.8 Hz, 1H), 7.81–7.72 (m, 3H), 7.62 (s, 1H), 7.44–7.29 (m, 4H), 7.19 (t, *J* = 8.0 Hz, 1H), 6.55 (dd, *J* = 8.1, 1.5 Hz, 1H), 4.93−4.86 (m, 1H), 4.50 (d, *J* = 9.4 Hz, 1H), 4.43–4.37 (m, 1H), 4.28–4.24 (m, 1H), 3.68–3.28 (m, 9H), 3.08–3.01 (m, 2H), 2.98–2.80 (m, 5H), 2.43 (s, 3H), 2.33 (t, *J* = 7.4 Hz, 2H), 2.28–2.19 (m, 1H), 2.12–2.04 (m, 1H), 2.02–1.96 (m, 1H), 1.81−1.74 (m, 1H), 1.65–1.56 (m, 2H), 1.55–1.32 (m, 12H), 1.31–1.17 (m, 16H), 0.91 (s, 9H). ^13^C NMR (126 MHz, DMSO) δ 172.46, 171.36, 171.02, 169.99, 156.27, 154.29, 151.89, 148.16, 145.11, 132.55, 131.56, 130.11, 129.25, 128.73, 127.80, 126.79, 124.88, 120.87, 117.65, 115.29, 112.67, 69.16, 59.01, 56.77, 56.65, 55.99, 51.42, 51.05, 48.06, 44.24, 42.23, 38.35, 38.07, 35.46, 35.23, 34.02, 33.71, 32.34, 31.75, 29.79, 29.06, 28.82, 26.67, 26.19, 25.63, 24.76, 23.12, 22.83, 22.47, 22.39, 16.38. LC/MS *m*/*z* calculated [M + H]^+^. 1176.58, found 1176.60.

### 3.3. Cloning, Expression, and Purification of SHP2 Protein

The SHP2 protein (Aa 1–528) was cloned into a pET-21a(+) vector. Bacterial BL21(DE3) (Novagen) was used as an expression host, and the induction of protein expression was carried out in LB media with 1 mM IPTG at 18 °C overnight. Cell pellets were stored at −80 °C for subsequent protein purification. Protein purification was conducted at 4 °C. Frozen cell pellets were lysed by sonication in 40 mL cold lysis buffer (50 mM Tris-HCl, pH 8.0, 150 mM NaCl, 5 mM imidazole, and 1 mM PMSF) per liter cell pellet. Cell lysates were clarified by centrifuging for 15 min at 6000 rpm. The supernatant was incubated with HisPur Ni-NTA resin (Thermo Scientific, Waltham, MA, USA) for 2 h, and then packed onto a column and washed with 50 resin volume of buffer A (50 mM Tris-HCl, pH 8.0, 500 mM NaCl, 5 mM imidazole). The HIS-tagged proteins were eluted with Buffer B (50 mM Tris-HCl, pH = 8.0, 500 mM NaCl, 300 mM imidazole,). Pooled HIS-protein-containing fractions were concentrated, loaded onto a HiLoad 26/600 Superdex 75 column (GE Healthcare Biosciences, Piscataway, NJ, USA), and eluted with storage buffer (50 mM Tris-HCl, pH 8.0, 150 mM NaCl, 1 mM DTT, 10% glycerol). Proteins used for inhibition assays were purified using Ni-NTA resin (Qiagen, Hilden, Germany) followed by size exclusion column chromatography (ÄKTA pure, Cytiva, Marlborough, MA, USA) and the purity was determined to be >95% by SDS-PAGE and Coomassie staining. The protein was aliquoted and stored at −80 °C.

### 3.4. SHP2 Allosteric Inhibition Assay and Determination of IC_50_ Values

The catalytic activity of SHP2 (1-528) was assayed with 6,8-Difluoro-4-Methylumbelliferyl Phosphate (DiFMUP, Invitrogen, cat# D6567) as a substrate in 3,3-Dimethylglutaric acid (DMG) buffer (50 mM DMG, pH 7.0, 1 mM EDTA, 18 mM NaCl, 0.01% Triton X-100) at 25 °C. To determine the IC_50_ values, the assays were performed in 96-well plates (Corning Costar 3915). Series diluted compounds were incubated with 0.5 nM of SHP2 and 0.5 μM of peptide IRS1_pY1172(dPEG8)pY1222 (sequence H2N-LN(pY)IDLDLV(dPEG8)LST(pY)ASINFQK-amide). After 30 min incubation at room temperature, the substrate DiFMUP was added to the reaction (200 μM final concentration, total reaction volume is 200 µM) and incubated at 25 °C for 10 min. The reaction was then quenched by adding 40 μL of 160 μM bpV(Phen) (Sigma–Aldrich, cat# SML0889) solution. The fluorescence signal was monitored with a CLARIOstar Plus Microplate Spectrophotometer (BMG Labtech) using excitation and emission wavelengths of 340 and 450 nm, respectively. The inhibitordose-response curves were analyzed using normalized IC_50_ regression curve fitting with control-based normalization. Data were fitted using Prism GraphPad 9.2.0.

### 3.5. PTP Inhibition Assay and Determination of IC_50_ Values

PTP activity was assayed using p-nitrophenyl phosphate (pNPP) as a substrate in DMG buffer (50 mM DMG, pH 7.0, 1 mM EDTA, 150 mM NaCl, 2 mM DTT, 0.1 mg/mL BSA) at 25 °C. The assays were performed in 96-well plates. To determine the IC_50_ values, the reaction was initiated by the addition of enzyme (final concentration = 10 nM) to a reaction mixture (0.2 mL) containing pNPP (at a final concentration close to the K_m_s of tested enzymes, in specific: 0.05 mM for CDC-14A; 0.5 mM for FAP-1; 2 mM for TC-PTP, PTP1B, STEP, LWM-PTP, and PTPα; 3 mM for SHP1; 4 mM for Laforin; 5 mM for LYP, CD45, and VHR; 6 mM for PTP-MEG2 and HePTP) with various concentrations of inhibitors. The reaction rate was measured using a SpectraMax Plus 384 Microplate Spectrophotometer (Molecular Devices, San Jose, CA, USA). Data were fitted using the SigmaPlot Enzyme Kinetics Module (Systat Software, Inc., San Jose, CA, USA).

### 3.6. Cell Culture, Western-Blot Analysis, and Colony Formation Assay

HEK293, SKBR3, U2OS, MCF7 and A549 cells were grown in DMEM supplemented with 10% FBS, penicillin (50 units/mL), and streptomycin (50 μg/mL) in a 37 °C incubator containing 5% CO_2_. KYSE-520 and H358 cells were grown in RPMI-1640 supplemented with 10% FBS, penicillin (50 units/mL), and streptomycin (50 μg/mL) in a 37 °C incubator containing 5% CO_2_. For western-blot analysis, protein samples were separated by SDS-PAGE, transferred to nitrocellulose membranes, and incubated overnight at 4 °C with corresponding primary antibodies diluted in 5% BSA in phosphate-buffered saline Tween (PBST). SuperSignal™ West Pico PLUS (PI34580; Thermo Scientific) was used to visualize the antibodies in the bioanalytical imaging system (Azure Biosystems c500). Anti-p-ERK1/2 (T202/Y204) (4370), and anti-ERK1/2 (4696) were purchased from Cell Signaling Technology. Anti-GAPDH (sc-365062), anti-SHP2 (sc-7384), anti-SHP1 (sc-7289), and anti-Actin (sc-8432) antibodies were purchased from Santa Cruz. Anti-PTP1B (ab244207) antibody was purchased from Abcam. For colony formation assay, a total of approximately 200–500 cells were seeded to each well in a 12-well plate. The wells were then treated with DMSO, compound **P9** and **3** at 37 °C for 3 weeks, gently washed, and stained with crystal violet.

### 3.7. Cell Proliferation Assay

The cell proliferation inhibition EC_50_ was determined by CCK-8 assay [40]. KYSE-520 cells were seeded at 5 × 10^3^ cells/well in 96 well plates. After 24 h, **P9** was added to the medium starting at 80 μM in a 2-fold dilution rate of 0.16 μM. After 7 days, 10 μL of CCK-8 test solution (APExBIO Technology, Houston, TX, USA) was added into each well and incubated for 2 h at 37 °C. The optical density (OD) at 450 nm was measured with a microplate reader (Molecular Device, Sunnyvale, CA, USA). The cell viability rate at different concentrations of **P9** treatment was determined with Prism, version 9.5.1 (GraphPad, San Diego, CA, USA).

### 3.8. Mouse Study

Experiments on mice were carried out in accordance with the regulations of the Institutional Animal Care and Use Committees at Purdue University. All mice were housed under pathogen-free conditions in the animal facility and received autoclaved water and food. Eight to ten weeks-old Nude mice were used in the study.

For pharmacokinetic studies, mice were administered a single dose of **P9** at 25 or 50 mg/kg via IP injection. Blood samples were collected through the tail vein at indicated time points after injection. Isoflurane was used as an anesthetic. All blood samples were centrifuged at 1500 g for 5 min, and plasma was separated and stored at –80 °C until analysis by a validated method based on reversed-phase liquid chromatography coupled to mass-spectrometric detection (LC/MS) using a previously published procedure [41].

For the xenograft tumor study, KYSE-520 cells were suspended in PBS and a total of 3 × 10^6^ cells (100 μL) were subcutaneously implanted into both the left and right flank using a 27-gauge needle. Tumor volume was calculated using the formula V = (W^2^ × L)/2 for caliper measurements. Once the tumor volume reaches 200 mm^3^, daily intraperitoneal injection of either control, 25 mg/kg or 50 mg/kg of compound **P9** was performed. Mice were sacrificed after injection for 18 days, and tumors were collected for biochemical analysis.

## 4. Conclusions

In summary, we have developed a new class of SHP2 degraders by recruiting a SHP2 allosteric inhibitor as the ligand and VHL as the E3 ubiquitin ligase. Thalidomide-based CRBN ligands were found incompatible with the allosteric inhibitors due to stability concerns. The linker length and composition were surveyed and compound **P9** was identified as the most potent candidate among this class of SHP2 degraders. Mechanistic studies demonstrated that **P9**-induced SHP2 degradation requires ternary complex formation and ubiquitin-proteasome participation. **P9** displayed a clear advantage over its parent SHP2 allosteric inhibitor in inhibiting the growth of a number of tumor cell lines including KYSE520. The in vivo studies indicate that **P9**, as a single agent, efficiently suppresses KYSE-520 xenograft tumor growth through SHP2 degradation and RAS/ERK1/2 inhibition. We believe that this SHP2 degrader could be used as a new chemical tool for further investigating the functions of SHP2 in physiological and pathological states. **P9** could also serve as an excellent starting point for the development of novel therapeutics targeting SHP2.

## Figures and Tables

**Table 1:** Degradation results of first generation SHP2 PROTACs constructed using compound **4** and a VHL ligand ^1^.

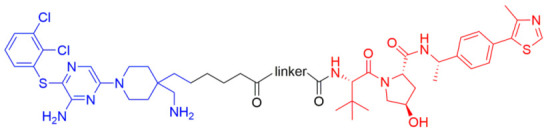				
Compound ID	Linker Structure	Linker Length (Atom)	Degradation at 1 μM	Degradation at 10 μM
**SC5**	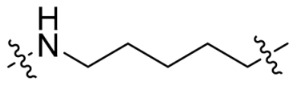	6	<10%	<10%
**SC7**	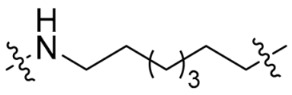	8	<10%	15%
**SC9**	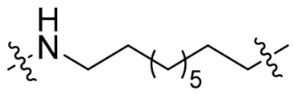	10	N.D. ^2^	25%
**SC11**	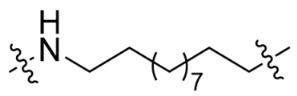	12	27%	64%
**P3**	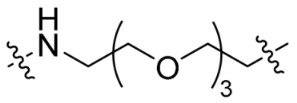	12	<10%	<10%
**P4**	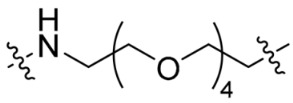	15	<10%	<10%
**P5**	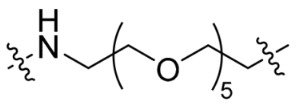	18	<10%	<10%

^1^ HEK293 cells, 16 h treatment. ^2^ N.D. = not determined.

**Table 2:** Degradation results of second generation SHP2 PROTACs constructed using compound **4** and a VHL ligand ^1^.

Compound ID	Linker Structure	Linker Length (Atom)	Degradation at 1 μM
**SC10**	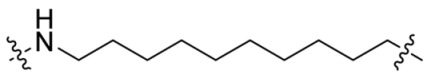	11	36%
**P7**	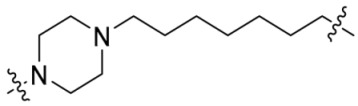	11	51%
**P8**	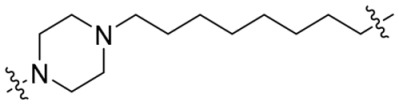	12	74%
**P9**	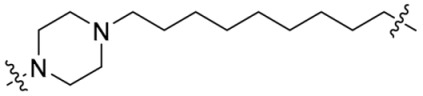	13	>95%
**P10**	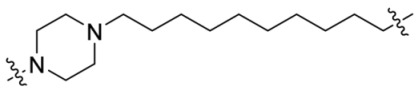	14	16%

^1^ HEK293 cells, 16 h treatment.

## Data Availability

Data are contained within the article and Supplementary Materials.

## Supplementary Materials

The following supporting information can be downloaded at: https://www.mdpi.com/article/10.3390/molecules28196947/s1, Figure S1: Western blots of degradation assay for PROTAC screening; Figure S2–S9: ^1^H and ^13^C NMR spectra of compound **6**, **10**, **L11**, and **P9**; Scheme S1: Decomposition of CRBN-based PROTACs in the presence of water; Table S1: Enzymatic IC_50_s of first generation SHP2 PROTACs against full length SHP2; Table S2: Enzymatic IC_50_s of second generation SHP2 PROTACs against full length SHP2; Table S3: Enzymatic IC_50_s of **P9** for a panel of 15 PTPs; Table S4: Plasma concentrations of **P9** in pharmacokinetic studies; Table S5: Pharmacokinetic analysis of **P9**.
