# Supplementary material for: Discovery of a SHP2 Degrader with In Vivo Anti-Tumor Activity
Source: Molecules. 2023 Oct 6;28(19):6947. doi: 10.3390/molecules28196947 (PMC10574094; doi:10.3390/molecules28196947)

Discovery of a SHP2 degrader with in vivo anti-tumor activity

Jinmin Miao[1], Yunpeng Bai[1], Yiming Miao[1], Zihan Qu[2], Jiajun Dong[1], Ruo-Yu Zhang[1], Devesh Aggarwal[1], Brenson A. Jassim[1], Quyen Nguyen[2], and Zhong-Yin Zhang[1,2,3,4*]

[1]Department of Medicinal Chemistry and Molecular Pharmacology, [2]Department of Chemistry, [3]Institute for Cancer Research, and [4]Institute for Drug Discovery, Purdue University, West Lafayette, IN 47907, USA,

[*]To whom correspondence should be addressed. Email: zhang-zy@purdue.edu

Table of Contents

1. Figure S1. Western blots of degradation assay for PROTAC screening ------------------------3
2. Figure S2. ^{1}H NMR spectra of intermediate **6** --4
3. Figure S3. ^{13}C NMR spectra of intermediate **6** ---4
4. Figure S4. ^{1}H NMR spectra of intermediate **10** --5
5. Figure S5. ^{13}C NMR spectra of intermediate **10** ---5
6. Figure S6. ^{1}H NMR spectra of intermediate **L11** ---6
7. Figure S7. ^{1}H NMR spectra of intermediate **L11** ---6
8. Figure S8. ^{1}H NMR spectra of PROTAC **P9** --7
9. Figure S9. ^{13}C NMR spectra of PROTAC **P9** ---7
10. Scheme S1. Decomposition of CRBN-based PROTACs in the presence of water ------------8
11. Table S1. Enzymatic IC_{50}s of first generation SHP2 PROTACs against full length SHP2------8
12. Table S2. Enzymatic IC_{50}s of second generation SHP2 PROTACs against full length SHP2--9
13. Table S3. Enzymatic IC_{50}s of **P9** for a panel of 15 PTPs ---9
14. Table S4. Plasma concentrations of **P9** (µM) in pharmacokinetic studies -------------------10
15. Table S5. Pharmacokinetic study data --10
16. Original images of unprocessed Western blots---11

Supplemental Figures, Schemes, and Tables:

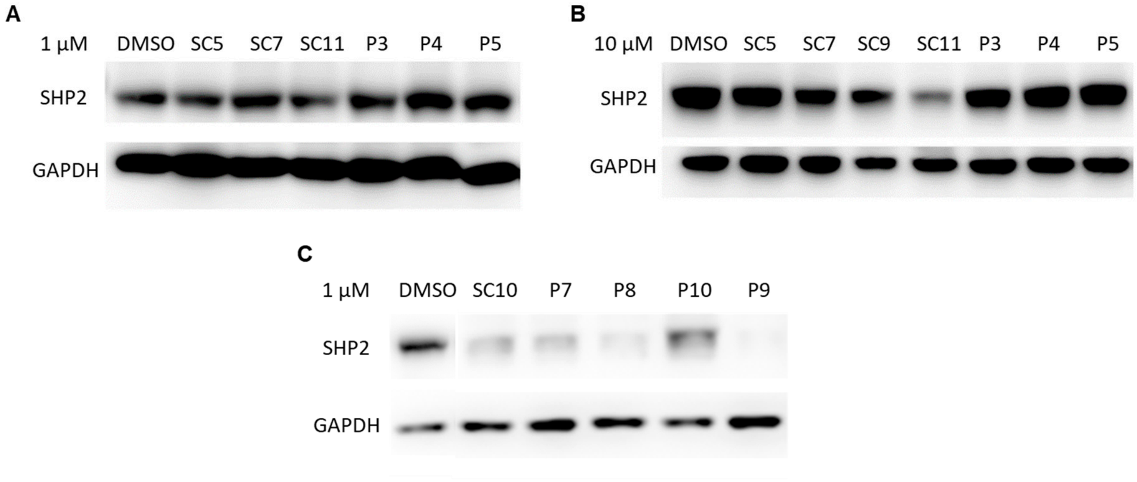

Figure S1. Western blots of degradation assay for PROTAC screening. (A) Degradation assay of compounds in Table 1 at 1 μM for 16 hours in HEK293 cells. (B) Degradation assay of compounds in Table 1 at 10 μM for 16 hours in HEK293 cells. (C) Degradation assay of compounds in Table 2 at 1 μM for 16 hours in HEK293 cells.

Figure S2. ¹H NMR spectra of intermediate 6

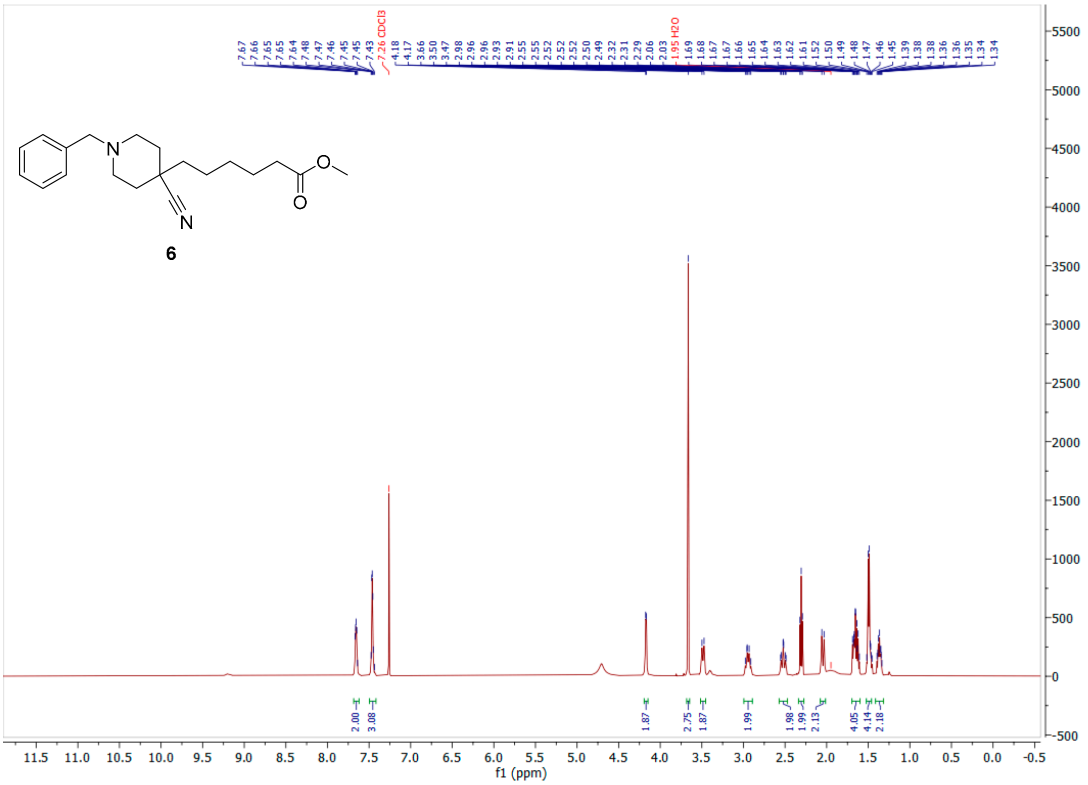

Figure S3. ¹³C NMR spectra of intermediate 6

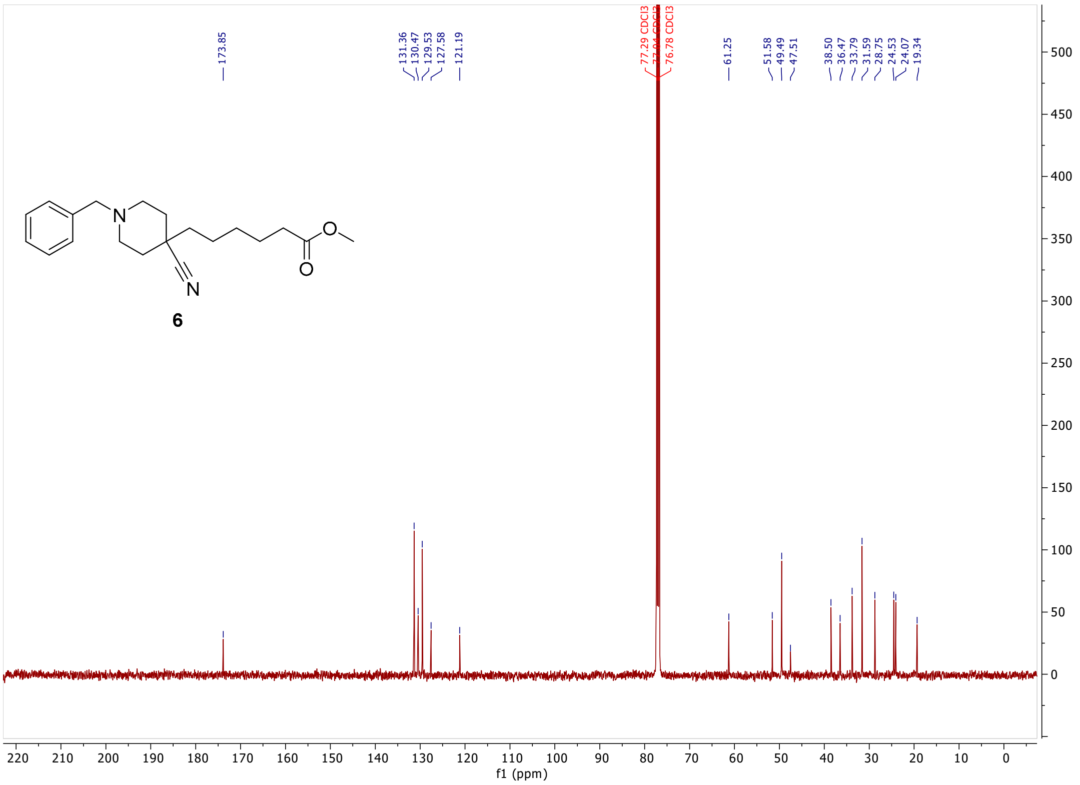

Figure S4. ^{1}H NMR spectra of intermediate 10

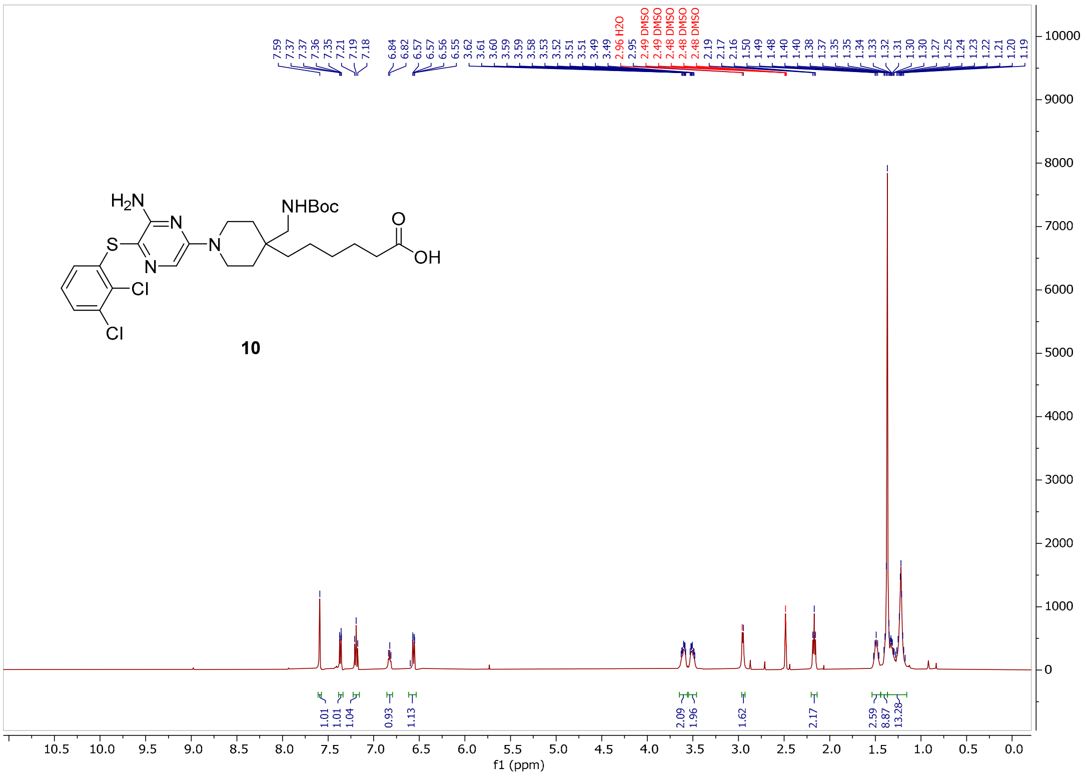

Figure S5. ^{13}C NMR spectra of intermediate 10

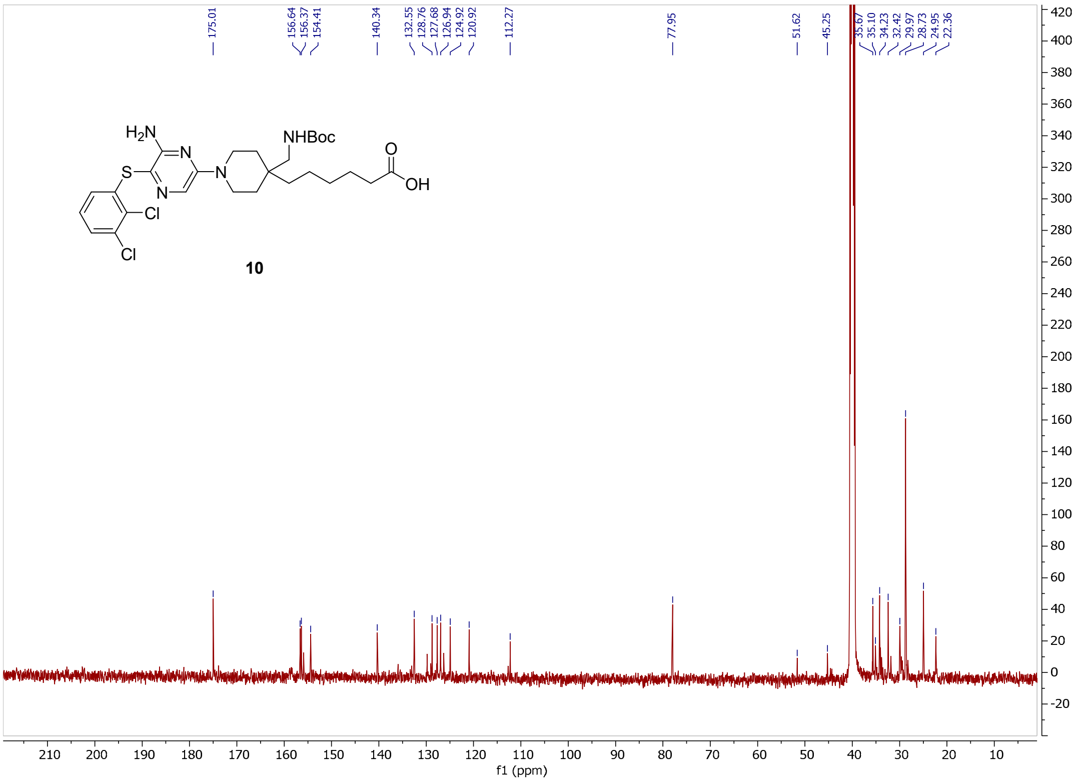

Figure S6. ^{1}H NMR spectra of intermediate L11

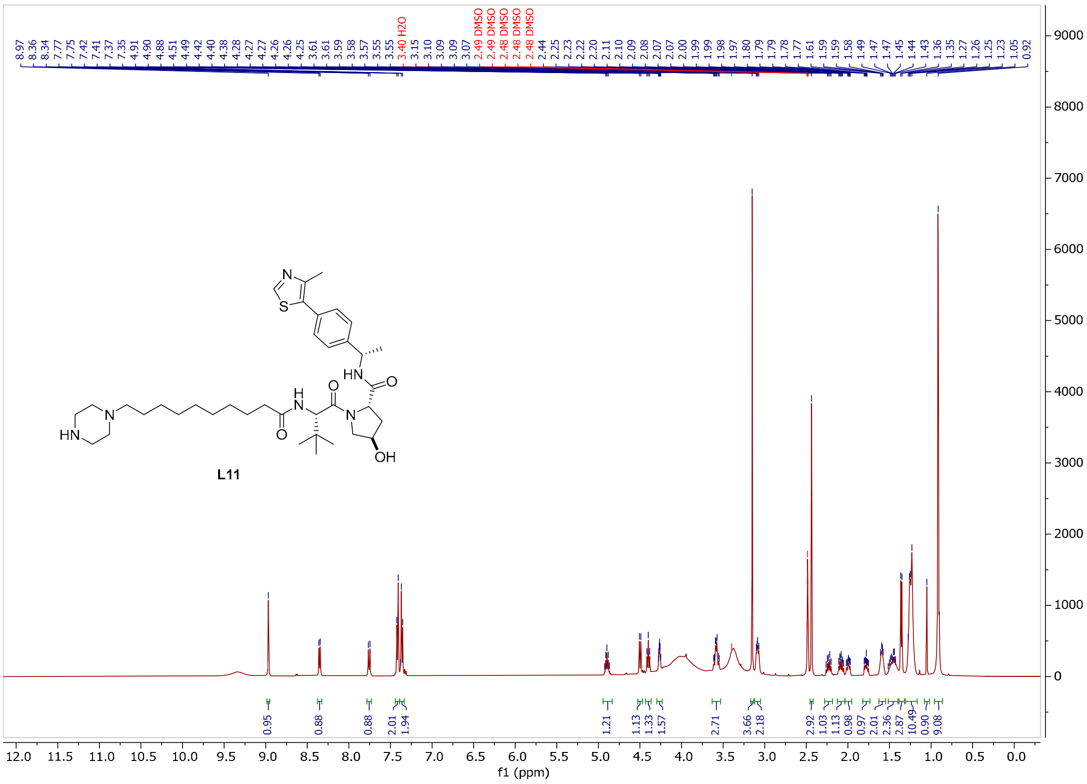

Figure S7. ^{1}H NMR spectra of intermediate L11

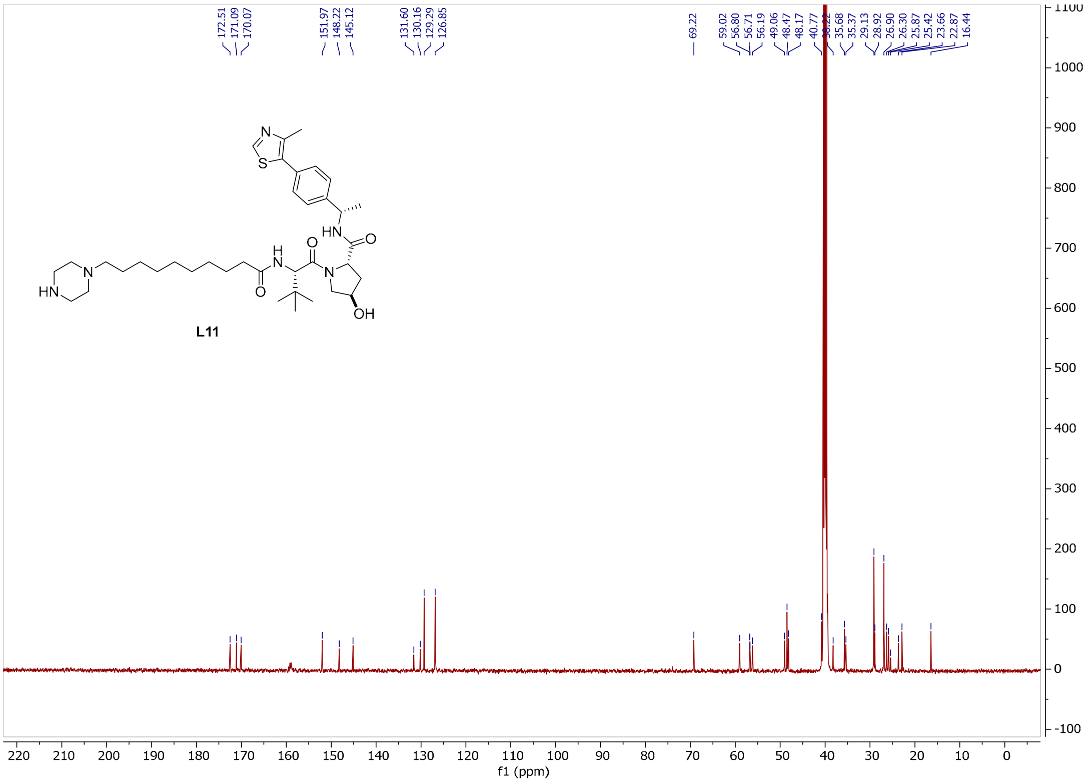

Figure S8. ^{1}H NMR spectra of PROTAC P9

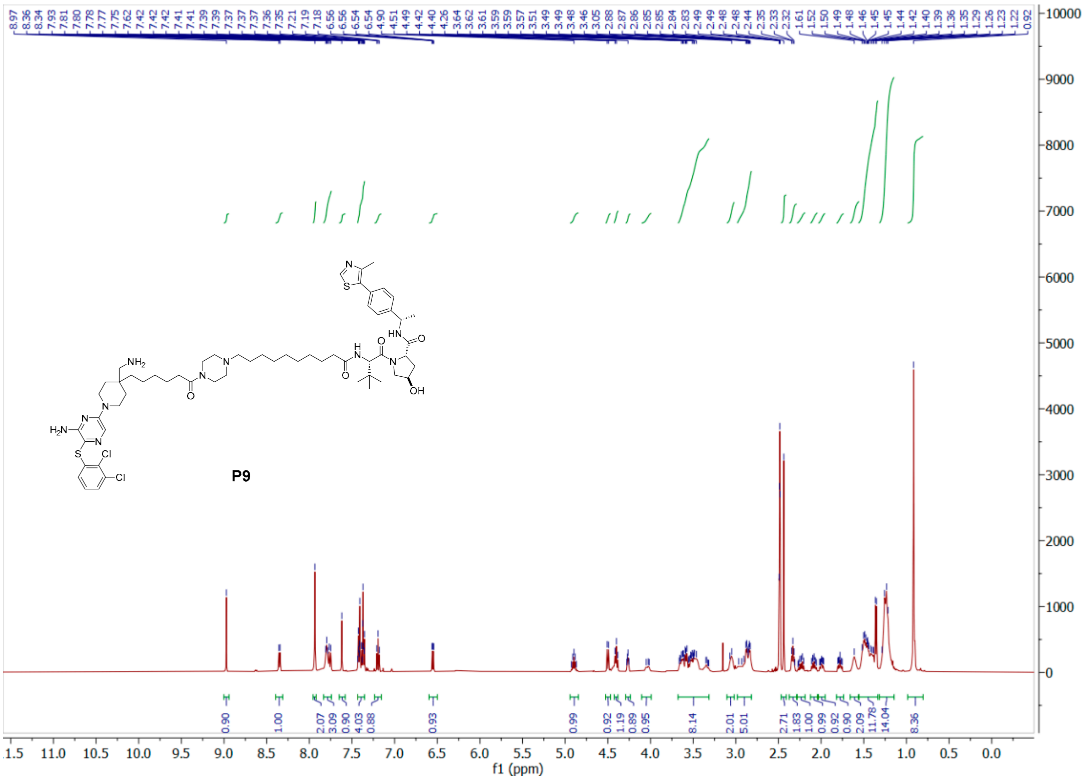

Figure S9. ^{13}C NMR spectra of PROTAC P9

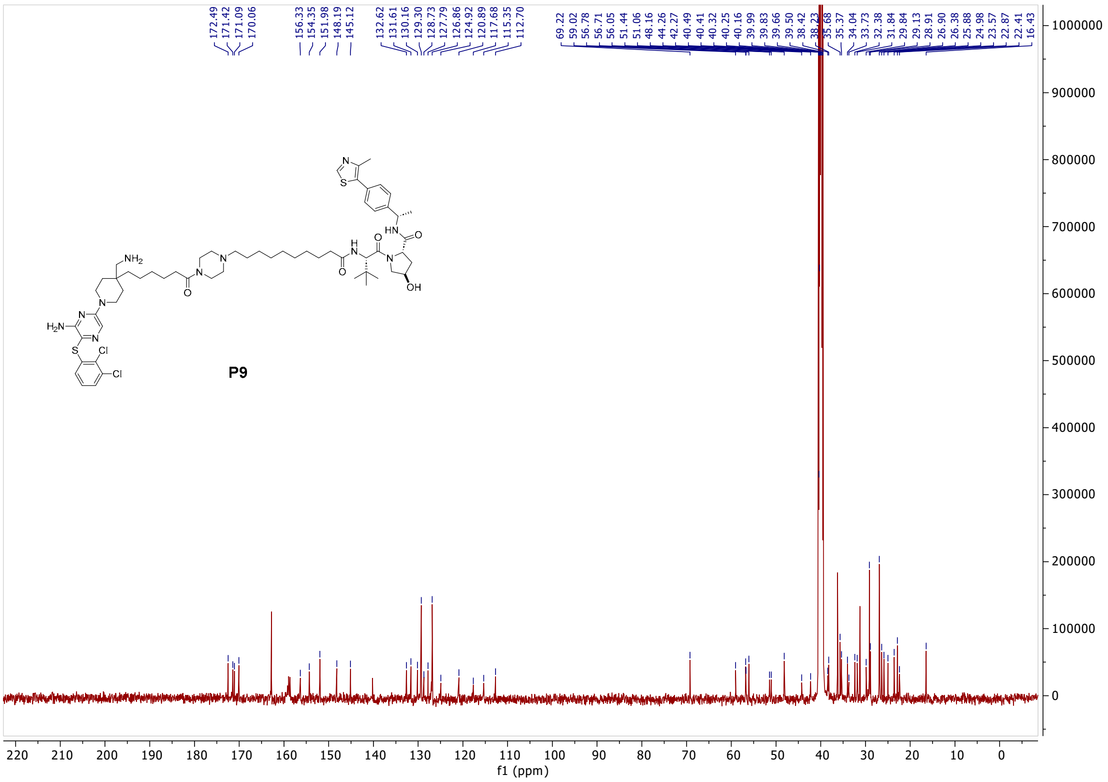

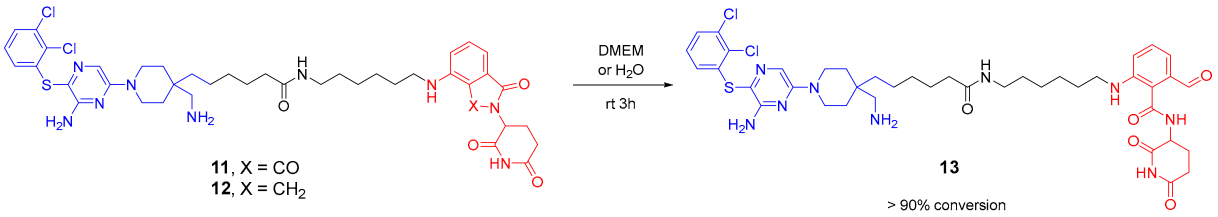

Scheme S1. Decomposition of CRBN-based PROTACs in the presence of water. 10 µM compound **11** or **12** was incubated in DMEM media or water for 3 hours. The final concentrations of the compounds at the endpoint were quantified by LCMS.

Table S1. Enzymatic IC_{50}s of first generation SHP2 PROTACs against full length SHP2

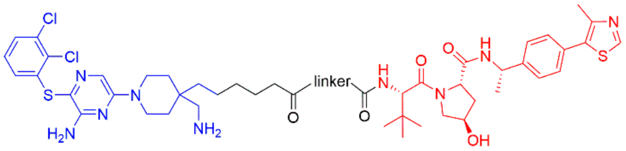

Compound ID	Linker Structure	Linker Length (atom)	SHP2 IC_{50} (nM)
SC5	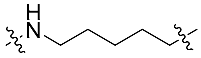	6	138.6 ± 10.4
SC7	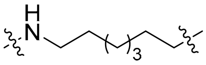	8	124.2 ± 21.6
SC9	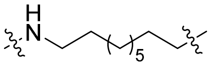	10	131.9 ± 8.7
SC11	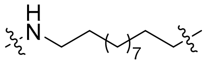	12	105.1 ± 15.6
P3	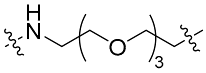	12	158.3 ± 26.5
P4	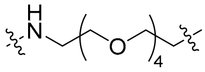	15	155.7 ± 15.8
P5	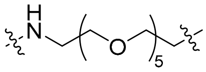	18	166.4 ± 11.9

Table S2. Enzymatic IC₅₀s of second generation SHP2 PROTACs against full length SHP2

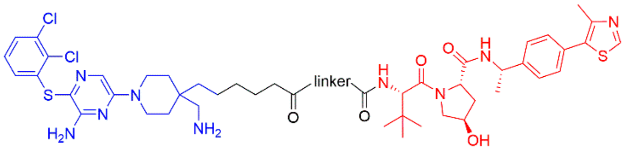

Compound ID	Linker Structure	Linker Length (atom)	SHP2 IC_{50} (nM)
SC10	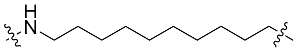	11	122.3 ± 19.1
P7	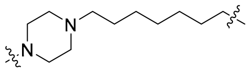	11	119.2 ± 25.4
P8	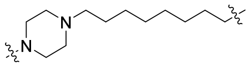	12	103.9 ± 9.3
P9	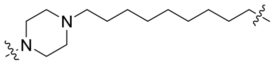	13	95.0 ± 3.4
P10	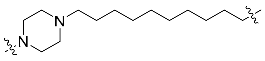	14	110.8 ± 8.2

Table S3. Enzymatic IC_{50}s of P9 for a panel of 15 PTPs

PTP	IC_{50}, μM	Fold selectivity
SHP2	0.095 ± 0.003	1
SHP1	>10	>105
PTP1B	>10	>105
TC-PTP	>10	>105
LYP	>10	>105
PTP-MEG2	>10	>105
HePTP	>10	>105
Laforin	>10	>105
STEP	>10	>105
LMW-PTP	>10	>105
CDC-14A	>10	>105
CD45	>10	>105
FAP-1	>10	>105
VHR	>10	>105
PTP-α	>10	>105

Table S4. Plasma concentrations of P9 (μM) in pharmacokinetic studies

Time (h)	25 mg/kg P9	25 mg/kg P9	25 mg/kg P9	50 mg/kg P9	50 mg/kg P9	50 mg/kg P9
0.5	0.56	0.52	0.75	1.76	1.82	1.96
1	1.02	1.2	1.32	2.51	2.7	2.55
2	0.92	1.09	0.93	1.66	1.76	1.7
3	0.55	0.62	0.59	1.03	1.22	1.15
6	0.36	0.41	0.39	0.55	0.7	0.68
24	0.13	0.18	0.06	0.16	0.24	0.1

Table S5. Pharmacokinetic analysis of P9

Dose	$t_{1/2}$ (h)	C_{max} (μM)	T_{max} (h)	AUC (μM*h)
25 mg/kg	3.71 ± 0.72	1.18 ± 0.13	2	8.51 ± 0.60
50 mg/kg	3.01 ± 0.48	2.51 ± 0.19	2	15.09 ± 0.99

Original images of unprocessed Western blots

Figure 3A

SHP2

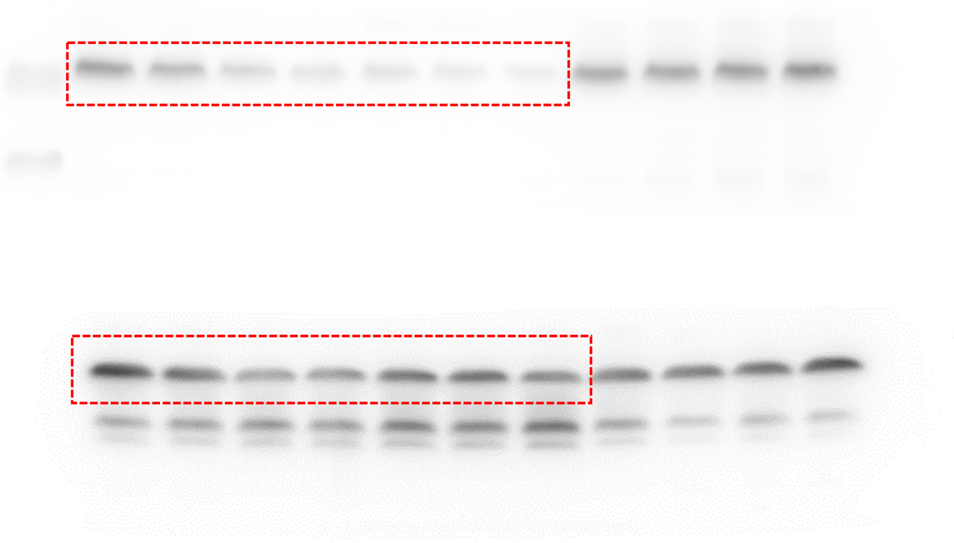

GAPDH

Figure 3B

SHP2

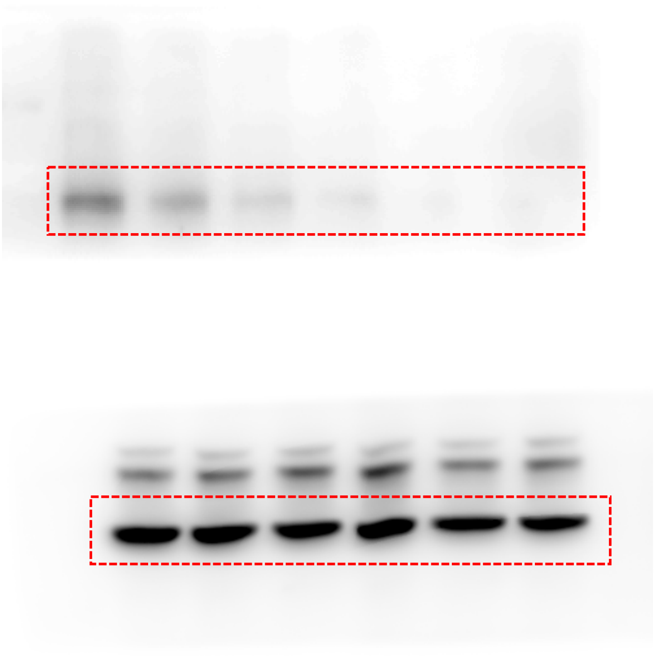

GAPDH

Figure 3C

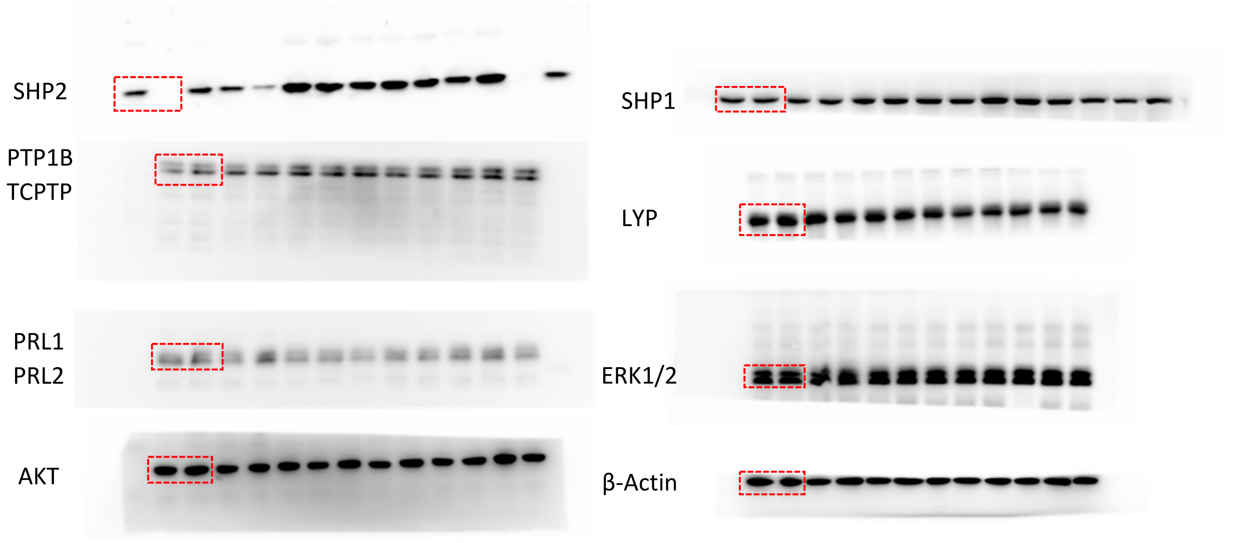

Figure 3D

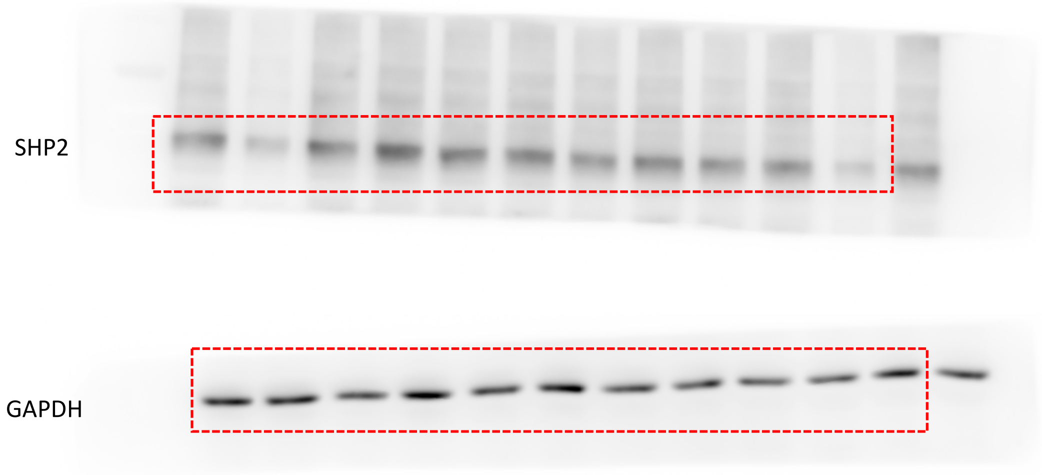

Figure 4A

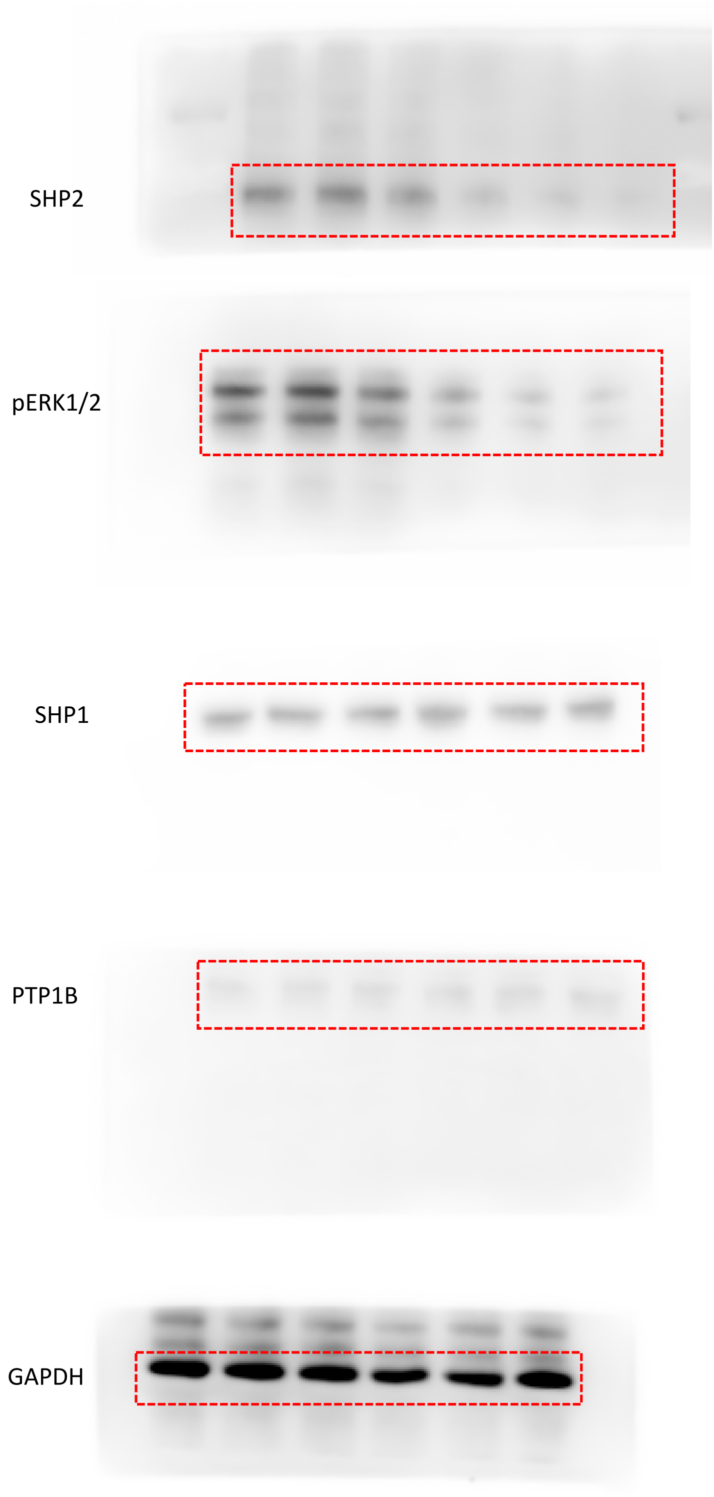

Figure 5D

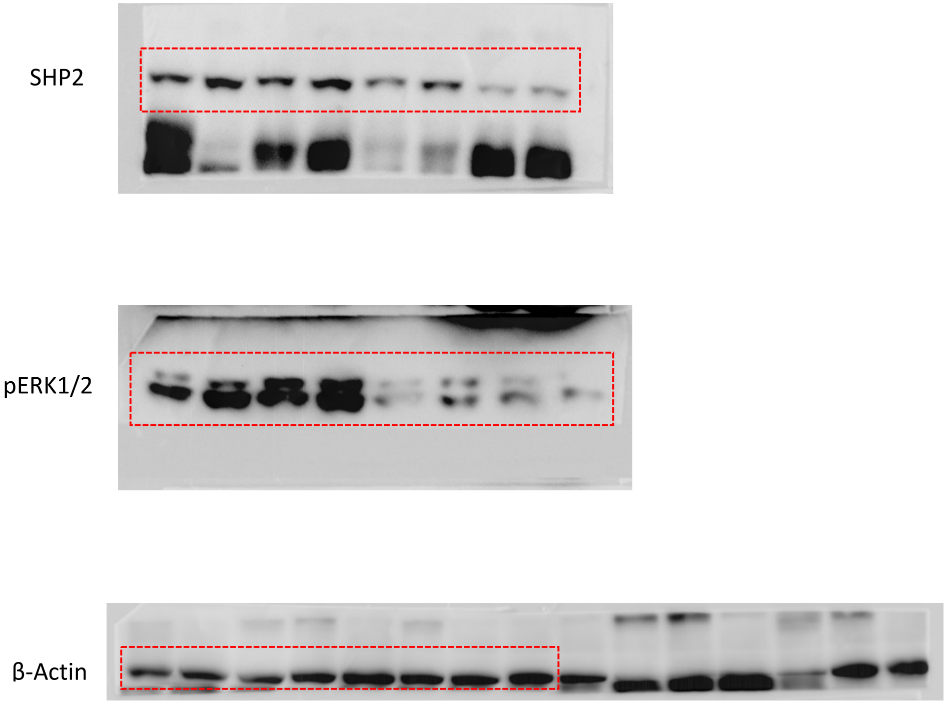

Figure S1A

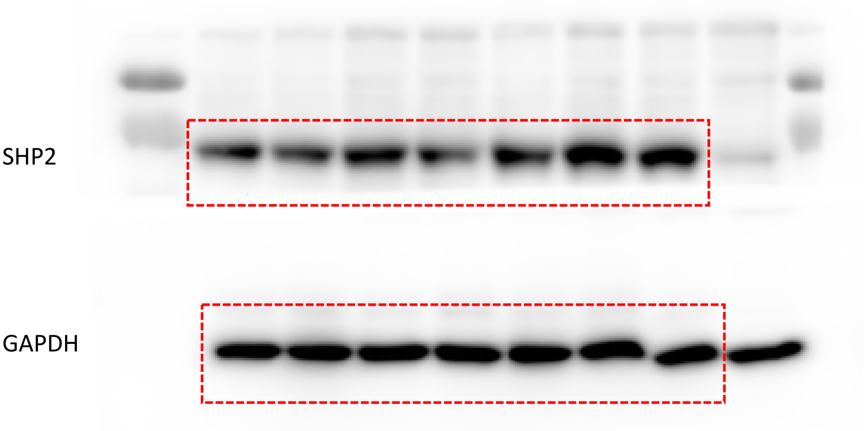

Figure S1B

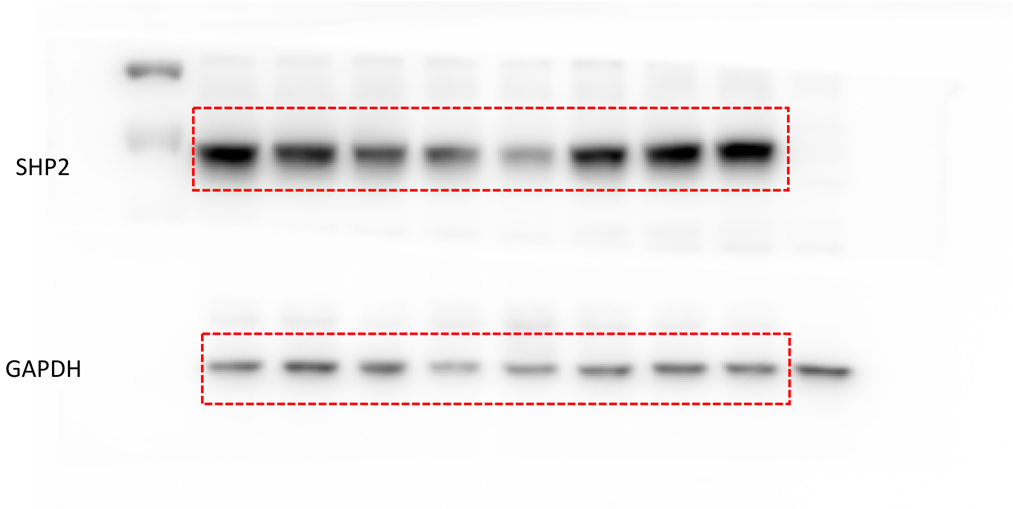

Figure S1C

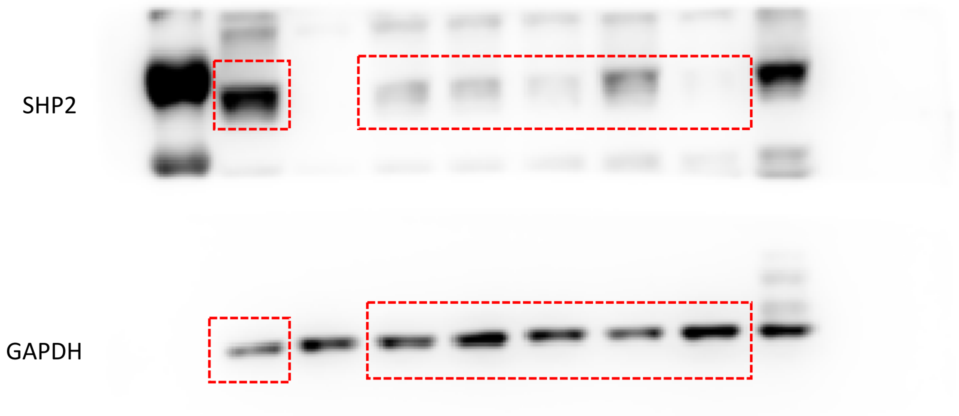

Supplement: Supplementary file 1 [file molecules-28-06947-s001.zip › molecules-2620717-SI.pdf]
